# The proton-sensing OGR1 receptor and hypoxia-inducible factors promote metal ion–induced inflammatory responses in coronary artery smooth muscle cells

**DOI:** 10.1016/j.jbc.2025.110842

**Published:** 2025-10-22

**Authors:** Koichi Sato, Chihiro Mogi, Haruka Aoki-Saito, Tamotsu Ishizuka, Jun Shirakawa, Hideaki Tomura, Dong-Soon Im

**Affiliations:** 1 Laboratory of Signal Transduction, Institute for Molecular and Cellular Regulation, Gunma University, Maebashi, Japan; 2 Laboratory of Diabetes and Metabolic Disorders, Institute for Molecular and Cellular Regulation, Gunma University, Maebashi, Japan; 3 Laboratory of Mucosal Ecosystem Design, Institute for Molecular and Cellular Regulation, Gunma University, Maebashi, Japan; 4 Department of Respiratory Medicine, Gunma University Graduate School of Medicine, Maebashi, Japan; 5 Department of Respiratory Medicine, Faculty of Medical Sciences, University of Fukui, Fukui, Japan; 6 Laboratory of Cell Signaling Regulation, Department of Life Sciences, School of Agriculture, Meiji University, Kawasaki, Japan; 7 College of Pharmacy, Kyung Hee University, Seoul, Republic of Korea

**Keywords:** acidic micromilieu, cell signaling, G protein–coupled receptor (GPCR), hypoxia-inducible factor (HIF), metal ion–protein interaction, vascular action

## Abstract

Divalent metal ions such as nickel, cobalt, and manganese are known to induce hypoxia-inducible factors (HIFs) over several hours and are implicated in inflammatory responses; however, their roles in vascular tissue remain incompletely understood. In addition to long-term effects, metal ions also can elicit rapid cellular responses, such as calcium mobilization and the phosphorylation of signaling molecules, within seconds to minutes. These rapid responses cannot be solely explained by HIF activation. Here, we investigated the contributions of both HIFs and proton-sensing ovarian cancer G protein–coupled receptor 1 (OGR1) to metal ion–induced inflammatory responses in human coronary artery smooth muscle cells. While metal ions induced HIF-α subunits and upregulated vascular endothelial growth factor a and leptin expression through relatively slow pathways, they simultaneously triggered the rapid induction of interleukin-6 and cyclooxygenase-2 *via* OGR1. Interleukin-6 secretion induced by metal ions and acidic pH was mediated through the OGR1/G_q/11_/Ca^2+^ pathway, including PKC, protein kinase D, and Ca^2+^/calcium/calmodulin-dependent protein kinase II, with a major contribution from the OGR1/G_q/11_/protein kinase D/CREB signaling axis. Furthermore, OGR1 could detect subtle changes in metal ion concentrations under mildly acidic conditions, suggesting a synergistic mechanism. We conclude that metal ions exert dual-phase inflammatory effects in vascular tissue: a rapid response *via* OGR1 signaling and a slower response *via* HIF-mediated transcription, both contributing to vascular inflammation.

Metal ions such as nickel, cobalt, and manganese are known to elicit allergic reactions. These ions inhibit the oxygen-dependent degradation of hypoxia-inducible factor (HIF) α subunits, leading to their stabilization and accumulation (1). As a result, HIF transcriptional activity increases, promoting the expression of genes associated with hypoxia responses (2, 3, 4, 5, 6, 7, 8, 9), angiogenic factors such as vascular endothelial growth factor a (VEGFa) and leptin, as well as inflammatory mediators including interleukin 6 (IL-6) and cyclooxygenase 2 (COX-2). While these effects represent relatively slow cellular responses occurring over several hours, metal ions have also been known to promote rapid cellular responses such as intracellular calcium mobilization and inositol phosphate production within seconds to minutes (10, 11, 12, 13). HIF alone cannot account for such rapid signaling events, implicating cell surface receptors in the regulation of these responses.

It is hypothesized that metal ions regulate inflammation *via* both rapid and delayed mechanisms. Although the precise physiological roles of metal ions in vascular tissues remain unclear, a prior report has demonstrated that nickel induced IL-6 expression *via* HIF-1α in endothelial cells (2). Additionally, nickel, commonly used in cardiovascular stents (14), can provoke allergic reactions through HIF-1α signaling in vascular smooth muscle cells (3). Angiogenesis, multiple layers of vascular remodeling, and inflammation—processes involving smooth muscle cell proliferation—are key contributors to neointimal hyperplasia (15). From an environmental medicine standpoint, exposure to cobalt and nickel is linked to occupational asthma, especially in electroplating workers (16). Thus, understanding metal ion–induced inflammatory responses is critical for both cardiovascular and respiratory health, with implications for the development of therapeutic targets.

The production of IL-6 is regulated in part by cAMP-responsive element-binding protein (CREB), which requires phosphorylation at S133 for activation (17, 18, 19). CREB phosphorylation is stimulated by cAMP/protein kinase A (PKA) signaling and growth factors (20). Ca^2+^/calcium/calmodulin-dependent protein kinase II (CaMKII) can both positively and negatively regulate CREB through phosphorylation at S133 and S142 (21). Additionally, protein kinase D (PKD), a downstream effector in Ca^2+^ signaling, has been implicated in the phosphorylation of CREB at S133 (22, 23). The mRNA-stabilizing factor ARID5A, which is involved in IL-6 expression, is also regulated *via* the cAMP/PKA/CREB pathway (24, 25, 26), suggesting that CREB signaling plays a crucial role in IL-6 production.

Proton-sensing OGR1-family G protein–coupled receptors (GPCRs), including ovarian cancer G protein–coupled receptor 1 (OGR1), GPR4, and T-cell death-associated gene 8 (TDAG8), respond to mildly acidic pH higher than 6.4 (27). Ludwig *et al*. reported that OGR1 and GPR4 sense extracellular acidification, resulting in the activation of the phospholipase C (PLC)/Ca^2+^ and adenylyl cyclase/cAMP signaling pathways through G_q/11_ and G_s_, respectively (28). Later, proton sensitivity was also reported for TDAG8 (29, 30). G2A (from G2 accumulation) is also classified as an OGR1 family receptor; however, the proton sensitivity of the receptor is very low (27). OGR1 family receptors are thought to be activated through the protonation of histidine residues in their extracellular domains, promoting conformational changes and coupling with G proteins (28, 31, 32). OGR1 has recently been reported to be activated by divalent metal ions including nickel, iron, zinc, cobalt, and manganese, likely through similar histidine-mediated mechanisms (33). Thus, extracellular protons and divalent metal ions may activate OGR1 through its extracellular domains (34, 35, 36, 37). Moreover, the metal ion–sensing domain of OGR1 partially overlaps, but is not identical to, its proton-sensing domain (35, 36), implying the possibility of receptor-level crosstalk and synergistic regulation.

In the present study, we used human coronary artery smooth muscle cells (CASMCs) to investigate metal ion–induced inflammatory signaling. We confirmed that nickel chloride (NiCl_2_), CoCl_2_, and MnCl_2_ induce HIF-1α and HIF-2α under normal oxygen conditions, leading to the delayed upregulation of VEGFa and leptin independent of extracellular acidification. In contrast, the same metal ions and mildly acidic pH rapidly triggered IL-6 and COX-2 induction through OGR1. IL-6 secretion by metal ions was mediated through the OGR1/G_q/11_/Ca^2+^ signaling axis including protein kinase C (PKC), PKD, and CaMKII, with the OGR1/G_q/11_/PKD2/CREB pathway showing a dominant contribution. Furthermore, OGR1 was sensitive to subtle changes in the extracellular concentration of metal ions and protons, supporting a synergistic regulatory model. These findings suggest that metal ions regulate inflammatory responses in vascular tissue through dual mechanisms: a rapid response *via* OGR1 and a slower transcriptional response through HIFs.

## Results

### NiCl_2_ induces inflammatory responses in human CASMCs *via* both rapid and delayed mechanisms

NiCl_2_ elicited inflammatory responses in CASMCs *via* both rapid and delayed mechanisms; nevertheless, the precise mechanisms involved have yet to be elucidated. Transition metals such as Ni^2+^ and Co^2+^ are known to inhibit the oxygen-dependent degradation of HIF-1α and HIF-2α by replacing Fe^2+^ at the Fe^2+^-binding site of prolyl hydroxylase domain–containing proteins, thereby stabilizing HIF-α subunits and promoting transcriptional responses under hypoxic conditions. The HIF-α subunits are critical regulators of cytokine and the growth factor gene expression involved in angiogenesis and vascular remodeling (38, 39). In vascular cells, hypoxic conditions modulate the expression of various HIF target genes, including IL-6 (2), COX-2 (7), VEGFa (3, 40, 41, 42, 43), and leptin (8, 9). These responses typically occur over 12 to 72 h, indicating a relatively delayed process.

**Figure 1:**
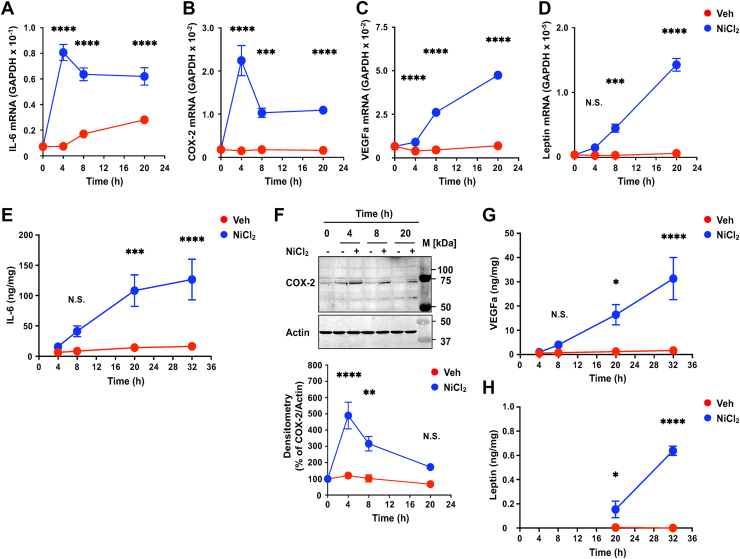
**NiCl_2_ induced inflammatory responses in human coronary artery smooth muscle cells *via* both rapid and relatively slower processes.***A*–*D*, CASMCs were serum starved in RPMI-1640-0.1% BSA for 8 h at 37 °C and incubated in RPMI-1640-Hepes (pH 7.4)-0.1% BSA with NiCl_2_ (300 μM, n = 6) or the vehicle (Veh, n = 6) for the times indicated (4, 8, and 20 h). Total RNA was prepared, and the mRNA expressions of IL-6 (*A*), COX-2 (*B*), VEGFa (*C*), and leptin (*D*) were analyzed by an RT-qPCR method. Results are expressed as relative ratios to GAPDH mRNA expression. Error bars represent the mean ± SEM. Comparisons of NiCl_2_*versus* the vehicle were assessed using a two-way ANOVA, followed by the Tukey test. The effect of NiCl_2_ was significant (∗∗∗*p* = 0.0002; ∗∗∗∗*p* < 0.0001), but that on leptin at 4 h was not significant (N.S.). *E*, extracellular IL-6 content was measured by the ELISA method after the serum-starved CASMCs were incubated with NiCl_2_ (300 μM) or the vehicle (Veh) for the times indicated (4, 8, 20, and 32 h). Results are expressed as ng/mg protein of adherent CASMCs. Error bars represent the mean ± SEM (n = 6). Comparisons of NiCl_2_*versus* the vehicle were assessed using a two-way ANOVA, followed by the Tukey test. The effect of NiCl_2_ was significant (∗∗∗*p* = 0.0005; ∗∗∗∗*p* < 0.0001), but that at 8 h was not significant (N.S. *p* = 0.4835). Although the overall comparison at 8 h did not reach statistical significance, there was an apparent trend toward increased IL-6 production. *F*, time course for the induction of COX-2 by NiCl_2_ in CASMCs. Serum-starved cells were incubated with NiCl_2_ (300 μM) or the vehicle (Veh) for the times indicated (4, 8, and 20 h). The COX-2 contents were measured by Western blotting. Gel images are representative results of three separate experiments. After quantification of COX-2 relative to actin, the results of densitometry are also expressed as percentages of the basal value (without incubation). Data are shown as the mean ± SEM of each group (n = 3) of NiCl_2_ and the vehicle (Veh). Comparisons of NiCl_2_*versus* the vehicle were assessed using a two-way ANOVA, followed by the Tukey test. The effect of NiCl_2_ was significant (∗∗∗∗*p* < 0.0001; ∗∗*p* = 0.0018), but that at 20 h was not significant (N.S. *p* = 0.1748). At 20 h, the overall comparison did not achieve statistical significance; however, there was a tendency for COX-2 production to increase. *G*, extracellular VEGFa content was measured by the ELISA method, after the serum-starved CASMCs were incubated with NiCl_2_ (300 μM) or the vehicle (Veh) for the times indicated (4, 8, 20, and 32 h). Other experimental procedures are the same as those described in (*E*). The effect of NiCl_2_ was significant ∗*p* = 0.0136; ∗∗∗∗*p* < 0.0001), but that at 8 h was not significant (N.S. *p* = 0.9459). *H*, extracellular leptin content was measured by the ELISA method, after the serum-starved CASMCs were incubated with NiCl_2_ (300 μM) or the vehicle (Veh) for the times indicated (20 and 32 h). Other experimental procedures are the same as those described in (*E*). The effect of NiCl_2_ was significant (∗*p* = 0.0294; ∗∗∗∗*p* < 0.0001). BSA, bovine serum albumin; CASMC, coronary artery smooth muscle cell; COX-2, cyclooxygenase; FSK, forskolin; VEGFa, vascular endothelial growth factor a; NiCl_2_, nickel chloride; IL-6, interleukin-6; RT-qPCR, quantitative real-time PCR.

To assess whether long-term exposure to NiCl_2_ affects the expression of IL-6, COX-2, VEGFa, and leptin in CASMCs, we stimulated cells at pH 7.4 for various durations (Fig. 1). Consistent with previous studies, relatively high concentrations of Ni^2+^ (0.5–1.5 mM) are often employed to analyze HIF-mediated responses. However, given the potential cytotoxicity of metal ions depending on concentration and exposure time, we used NiCl_2_ concentrations of up to 300 μM. NiCl_2_ induced a rapid increase in IL-6 and COX-2 mRNA levels, peaking at 4 h and declining thereafter (Fig. 1, *A* and *B*, respectively). In contrast, VEGFa and leptin mRNA levels showed delayed responses, increasing noticeably from 20 h after stimulation (Fig. 1, *C* and *D*, respectively). In a similar manner, COX-2 peptide accumulated intracellularly concomitant with its mRNA expression. By contrast, IL-6 peptide release occurred with a delay relative to its mRNA induction, first detectable in the culture medium at 8 h and reaching significant levels at 20 to 32 h (Fig. 1, *E* and *F*, respectively). In agreement with our previous findings in bronchial smooth muscle cells (44), IL-6 secretion induced by NiCl_2_ was clearly detectable in CASMCs, and subsequent experiments assessing IL-6 release were performed at 20 h. Thereafter, with an additional delay, the release of VEGFa and leptin became distinctly apparent at approximately 32 h (Fig. 1, *G* and *H*, respectively). These data suggest that NiCl_2_ triggers both rapid and delayed cellular responses in CASMCs, mediated *via* distinct signaling pathways.

### NiCl_2_, CoCl_2_, and MnCl_2_ induce HIF-1α and HIF-2α protein accumulation in human CASMCs

**Figure 2:**
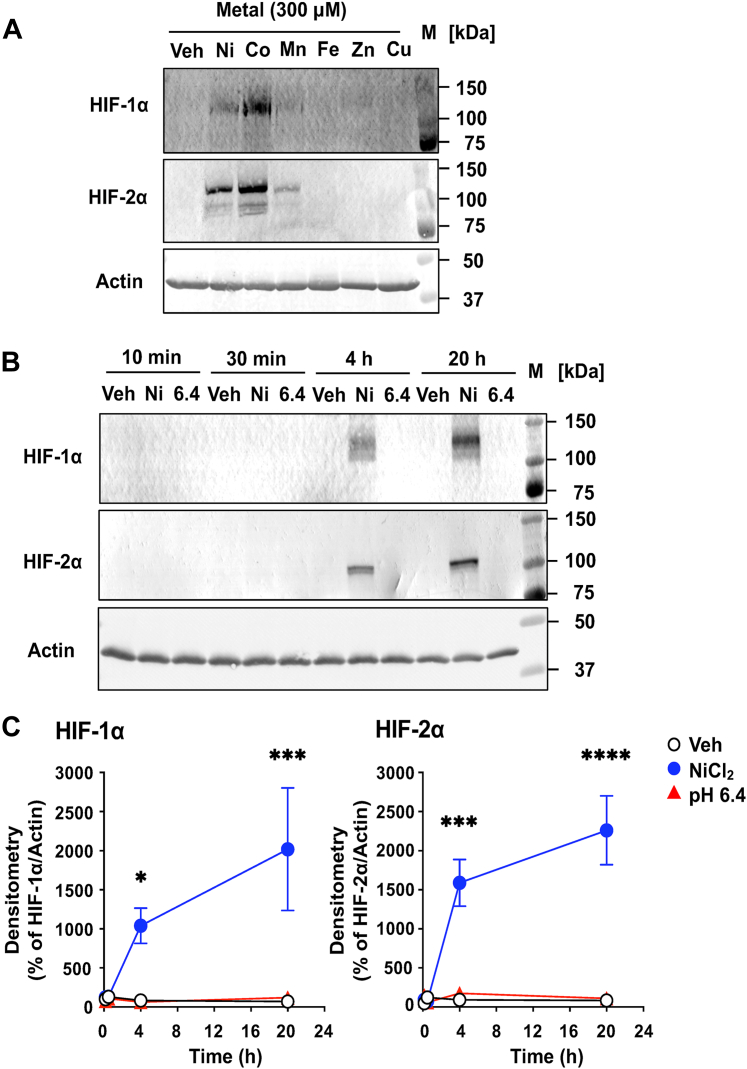
**NiCl_2_, CoCl_2_, and MnCl_2_ can mimic hypoxic conditions and lead to the induction of HIF-1α and HIF-2α in CASMCs.***A*, the effect of metal chlorides on the enhancements of the hypoxia-inducible transcription factors in CASMCs, even in the presence of oxygen. The cells were incubated at 37 °C for 20 h in RPMI-1640-Hepes (pH 7.4)-0.1% BSA with the indicated metal chlorides (300 μM) or the vehicle (Veh). The HIF-1α and HIF-2α contents were measured in cell lysate by Western blotting, as described in the Experimental procedures section. Gel images are representative results of three separate experiments (*A*). *B* and *C*, time course for the induction HIF-1α and HIF-2α by NiCl_2_ in CASMCs. The cells were incubated at 37 °C for the time indicated (10–30 min and 4–20 h) in RPMI-1640-Hepes (pH 7.4)-0.1% BSA with NiCl_2_ (300 μM), pH 6.4 (adjusted with 1 M HCl), or the vehicle (Veh). The HIF-1α and HIF-2α contents were measured by Western blotting. Gel images are representative results of three separate experiments (*B*). After quantification of HIF-1α and HIF-2α relative to actin, the results of densitometry are also expressed as percentages of the basal value. Data are shown as the mean ± SEM of each group (n = 3) of NiCl_2_ and the vehicle (Veh). Comparisons of NiCl_2_ or pH 6.4 *versus* the vehicle were assessed using a two-way ANOVA, followed by the Tukey test. The effect of NiCl_2_ was significant for HIF-1α (∗*p* = 0.0405; ∗∗∗*p* = 0.0009) and for HIF2α (∗∗∗*p* = 0.0002; ∗∗∗∗*p* < 0.0001). BSA, bovine serum albumin; CASMC, coronary artery smooth muscle cell; HIF, hypoxia-inducible factor; NiCl_2_, nickel chloride.

Using Western blotting, we next examined whether NiCl_2_ and metal chlorides induce HIF-1α and HIF-2α protein accumulation in CASMCs (Fig. 2). No induction was observed with FeCl_2_, ZnCl_2_, or CuCl_2_ (Fig. 2*A*). In contrast, both NiCl_2_ and CoCl_2_ robustly increased HIF-1α and HIF-2α levels at 20 h (Fig. 2*A*). Although MnCl_2_ showed no significant effect, a trend toward HIF accumulation was evident. Notably, FeCl_2_ triggered a transient [Ca^2+^]_i_ response (∼30 s) similar to that observed with NiCl_2_, CoCl_2_, and MnCl_2_ (Fig. 5), but failed to induce HIF accumulation at 4 h, likely due to Fe^2+^-mediated reactive oxygen and cytotoxicity. Dose-response studies showed that HIF accumulation was detectable with NiCl_2_ at 30 μM, peaking at 100 to 300 μM (Fig. S1*A*). The induction of HIF-1α and HIF-2α by NiCl_2_ and the following signaling processes remain unchanged under acidic conditions (Fig. S1, *A* and *B*, respectively).

Time-course analysis revealed that NiCl_2_-induced HIF-1α and HIF-2α protein levels were detectable at 4 h, increasing steadily up to 20 h (Fig. 2, *B* and *C*, respectively), which is consistent with previous studies (2, 3). Thus, in CASMCs, the accumulation of HIF-1α and HIF-2α by metal ions represents a slower response, paralleling the delayed mRNA expression of VEGFa and leptin.

### HIFα-subunit knockdowns do not affect NiCl_2_-induced IL-6 expression or secretion

**Figure 3:**
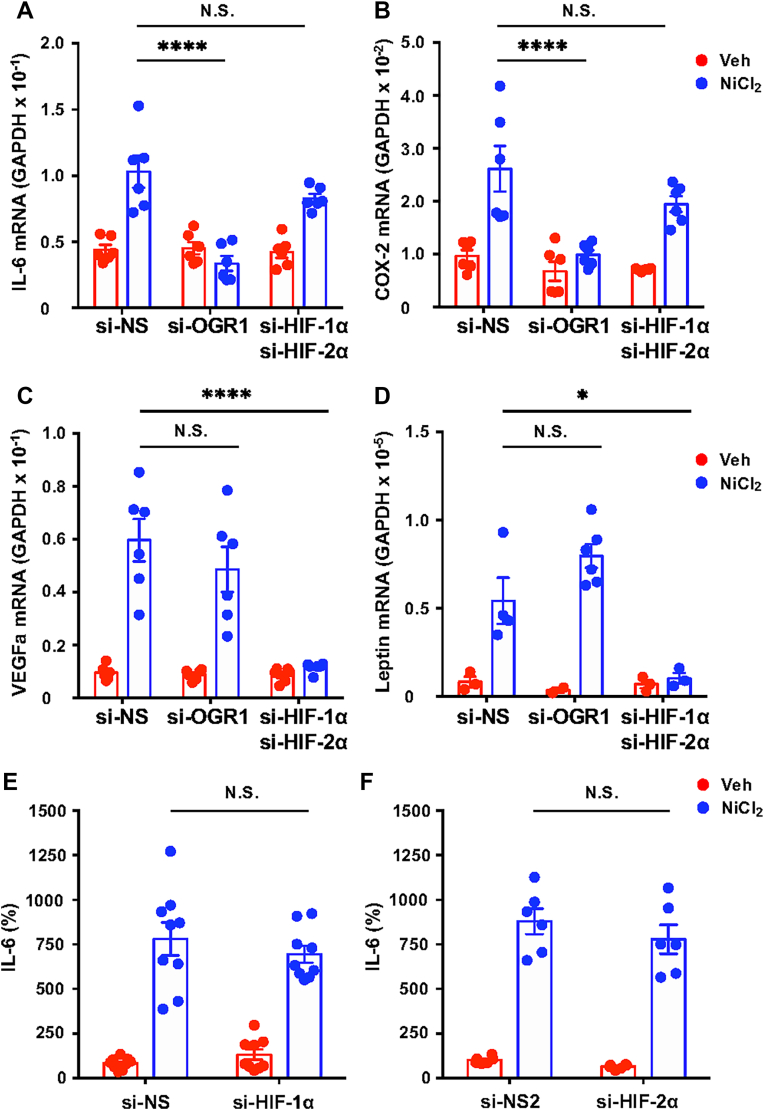
**NiCl_2_-mediated inflammatory responses through the activation of OGR1 and the induction of HIF proteins in CASMCs.***A*–*D*, effect of siRNAs on NiCl_2_-induced gene expression. After serum starvation for 8 h, CASMCs were incubated at 37 °C for 20 h in RPMI-1640-Hepes (pH 7.4)-0.1% BSA with NiCl_2_ (300 μM) or the vehicle (Veh). IL-6 (*A*), COX-2 (*B*), VEGFa (*C*), and leptin (*D*) were assessed by the RT-qPCR method. Results are expressed as relative ratios to GAPDH mRNA expression. Error bars represent the mean ± SEM (n = 6). Comparisons of siRNAs specific to OGR1 (si-OGR1) or HIF1A (si-HIF-1α) and EPAS1 (si-HIF-2α) *versus* control siRNA (si-NS) were assessed using a two-way ANOVA, followed by the Tukey test. The NiCl_2_-induced expression of IL-6 (*A*) and COX-2 (*B*) was significantly attenuated by si-OGR1 (∗∗∗∗*p* < 0.0001); however, si-HIF-1α and si-HIF-2α had no significant effect (N.S. *p* = 0.2538 or N.S. *p* = 2373, respectively). In contrast, the NiCl_2_-induced expression of VEGFa (*C*) and leptin (*D*) was markedly suppressed by si-HIF-1α and si-HIF-2α (∗∗∗∗*p* < 0.0001), while the effect of si-OGR1 was minimal (N.S. *p* = 0.5972 or N.S. *p* = 1682, respectively). *E* and *F*, the effect of siRNAs on NiCl_2_-induced IL-6 protein secretion. The serum-starved CASMCs were stimulated at 37 °C for 20 h by NiCl_2_ (100 μM) or the vehicle (Veh) in RPMI-1640-Hepes (pH 7.4)-0.1% BSA. IL-6 proteins were measured by an ELISA method. Results are expressed as percentages of the basal value obtained with the vehicle of si-NS cells. Error bars represent the mean ± SEM (n = 6). Comparisons of si-HIF-1α or si-HIF-2α *versus* si-NS were assessed using a two-way ANOVA, followed by the Tukey test. The effect of si-HIF-1α or si-HIF-2α on the NiCl_2_-induced IL-6 was not significant (N.S. *p* = 0.6832 or N.S. *p* = 0.5680, respectively). BSA, bovine serum albumin; CASMC, coronary artery smooth muscle cell; COX-2, cyclooxygenase; IL-6, interleukin-6; HIF, hypoxia-inducible factor; NiCl_2_, nickel chloride; OGR1, ovarian cancer G protein–coupled receptor 1; RT-qPCR, quantitative real-time PCR; VEGFa, vascular endothelial growth factor a.

To delineate the contributions of HIF and OGR1, siRNAs targeting HIF-1α, HIF-2α, and OGR1 were employed. Transfection with si-HIF-1α and si-HIF-2α achieved approximately 90% knockdown of their respective mRNAs (Fig. S2, *A* and *B*, respectively), and the induction of HIF proteins by NiCl_2_ or CoCl_2_ was barely detectable (Fig. S2*C*). Among the OGR1 family GPCRs, only OGR1 was detected in CASMCs, and its mRNA expression was reduced by ∼90% following si-OGR1 transfection (Fig. S3*A*). Under these conditions, metal chloride-induced OGR1 downstream responses, including Ca^2+^ mobilization and IL-6 production, were markedly suppressed to basal levels, as described below. Notably, the induction of HIF proteins by NiCl_2_ or CoCl_2_ was largely unaffected by si-OGR1 (Fig. S3*B*). OGR1 knockdown markedly reduced NiCl_2_-induced IL-6 and COX-2 mRNA expression (Fig. 3, *A* and *B*, respectively) but had no effect on VEGFa or leptin mRNA (Fig. 3, *C* and *D*, respectively). In contrast, HIF-α knockdown significantly decreased VEGFa and leptin mRNA levels (Fig. 3, *C* and *D*, respectively), but it had minimal impact on IL-6 or COX-2 expression (Fig. 3, *A* and *B*, respectively) and it did not alter IL-6 protein secretion (Fig. 3, *E* and *F*). These results suggest that NiCl_2_ seems to trigger IL-6 production *via* an OGR1-dependent pathway distinct from HIF signaling.

### IL-6 secretion is triggered by metal ions through OGR1 independent of the intracellular accumulation of cAMP

**Figure 4:**
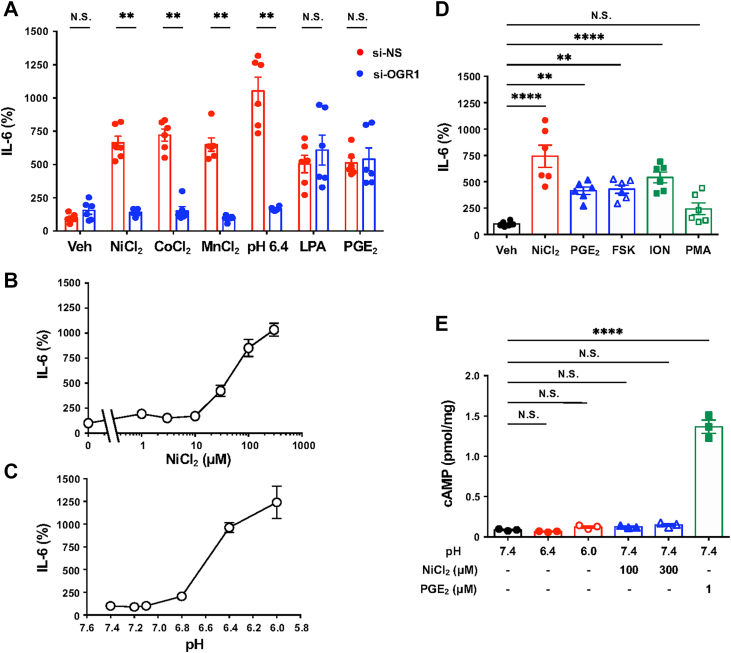
**IL-6 production in CASMCs as a result of the OGR1-mediated action by NiCl_2_ or pH independently of the intracellular accumulation of cAMP.***A*, effect of si-OGR1 on IL-6 secretion. The siRNA-transfected CASMCs were serum starved for 8 h and stimulated at 37 °C for 20 h in RPMI-1640-hepes (pH 7.4)-0.1% BSA containing NiCl_2_ (100 μM), CoCl_2_ (100 μM), MnCl_2_ (100 μM), pH 6.4 (adjusted with 1 M HCl), lysophosphatidic acid (LPA, 1 μM), or prostaglandin E2 (PGE_2_, 1 μM). IL-6 proteins were measured by the ELISA method. Results are expressed as percentages of the basal value obtained with the vehicle in si-NS cells. Error bars represent the mean ± SEM (n = 6). Comparisons of siRNAs specific to OGR1 (si-OGR1) *versus* control siRNA (si-NS) were assessed using a two-way ANOVA, followed by the Tukey test. The effect of siRNAs on metal chloride or pH 6.4 action was significant (∗∗*p* < 0.01); however, that of LPA or PGE_2_ action was not significant (N.S.). *B*, the dose-response curve of NiCl_2_-induced IL-6 secretion. The serum-starved CASMCs were stimulated at 37 °C for 20 h by the indicated concentrations of NiCl_2_ (1, 3, 10, 30, 100, and 300 μM) in RPMI-1640-Hepes (pH 7.4)-0.1% BSA. Other experimental procedures are the same as those described in (A). Results are expressed as percentages of the basal value obtained with the vehicle at pH 7.4. Error bars represent the mean ± SEM (n = 3). *C*, extracellular pH-dependent IL-6 secretion. CASMCs were incubated at 37 °C for 20 h in RPMI1640-Hepes (pH 7.4, 7.2 7.0, 6.8, 6.4, and 6.0)-0.1% BSA. Other experimental procedures are the same as those described in (*A*). Error bars represent the mean ± SEM (n = 3). *D*, the activation of the cAMP pathway and the Ca^2+^ signaling pathway plays a significant role in IL-6 production in CASMCs. The IL-6 secretion response to NiCl_2_ (100 μM), PGE_2_ (1 μM), forskolin (FSK, 1 μM), ionomycin (ION, 1 μM), or phorbol-12-myristate-13-acetate (PMA, 1 μM) was examined. Other experimental procedures are the same as those described in (*A*). Data are shown as the mean ± SEM of each group (n = 6). Comparisons of each test reagent *versus* the vehicle were assessed using a one-way ANOVA, followed by the Tukey test. As compared with the vehicle, the effects of the test reagents were significant for NiCl_2_ (∗∗∗∗*p* < 0.0001), PGE_2_ (∗∗*p* = 0.0023), FSK (∗∗*p* = 0.0014), and ION (∗∗∗∗*p* < 0.0001), whereas PMA showed no significant difference (N.S. *p* = 0.2826). Although the overall comparison did not reach statistical significance for PMA, it exhibited a trend toward increased IL-6 production, and an independent Student’s *t*-test revealed a significant difference between PMA and the vehicle (*p* < 0.05). (*E*) The intracellular cAMP accumulation response to acidic pH (6.4–6.0), NiCl_2_ (100 μM), or PGE_2_ (1 μM) was examined as described in the Experimental procedures section. CASMCs were incubated at 37 °C for 30 min in a Hepes-buffered medium containing 3-isobutyl-1-methylxanthine (IBMX, 0.5 mM) under the indicated reagent. Data are shown as the mean ± SEM of each group (n = 3). Comparisons of each test reagent *versus* the vehicle were assessed using a one-way ANOVA, followed by the Tukey test. cAMP responses were robustly induced by PGE_2_ (∗∗∗∗*p* < 0.0001), whereas neither pH 6.4 (N.S. *p* = 0.9927), pH 6.0 (N.S. *p* = 0.9117), 100 μM NiCl_2_ (N.S. *p* = 0.8948), or 300 μM NiCl_2_ (N.S. *p* = 0.5981) elicited a significant effect as compared with the basal level. BSA, bovine serum albumin; CASMC, coronary artery smooth muscle cell; HIF, hypoxia-inducible factor; IL-6, interleukin-6; NiCl_2_, nickel chloride; OGR1, ovarian cancer G protein–coupled receptor 1.

To determine whether metal chlorides stimulate IL-6 secretion, CASMCs were exposed to NiCl_2_, CoCl_2_, or MnCl_2_ for 20 h (Fig. 4*A*). All three metals, as well as a pH value of 6.4, lysophosphatidic acid (LPA), and prostaglandin E_2_ (PGE_2_), increased IL-6 secretion after stimulation for 20 h. In CASMCs, LPA and PGE_2_ are known to stimulate the LPAR1/G_q/11_/Ca^2+^ (45) and EP2 receptor/G_s_/cAMP signaling pathways, respectively. OGR1 knockdown specifically abolished the responses to metal chlorides but not to LPA or PGE_2_. Dose-response studies showed that IL-6 production was detectable with NiCl_2_ at 10 μM, peaking at 100 to 300 μM (Fig. 4, *B* and *C*). Acidic pH also induced IL-6 production, with a maximal effect at pH 6.4 to 6.0. When overexpressed, OGR1 is known to interact with both the G_s_/cAMP and G_q/11_/Ca^2+^ signaling pathways (46). The IL-6 production response in CASMCs was mimicked by ionomycin (ION, a selective Ca^2+^ ionophore), phorbol 12-myristate 13-acetate (PMA, a potent activator of PKC), PGE_2_, and forskolin (FSK, an adenylate cyclase activator), with reduced PMA efficacy, potentially due to desensitization (Fig. 4*D*). Although PGE_2_ markedly increased intracellular cAMP, NiCl_2_ and acidic pH did not (Fig. 4*E*). These results indicate that IL-6 induction by metal chlorides involves OGR1-mediated G_q/11_/Ca^2+^ signaling rather than G_s_/cAMP.

### OGR1 mediates metal-induced intracellular Ca^2+^ mobilization in CASMCs

**Figure 5:**
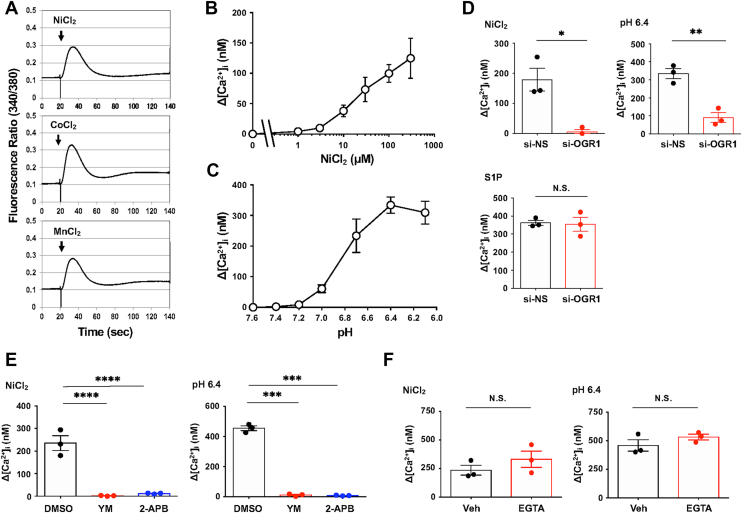
**[Ca^2+^]_i_ responses to acidic pH or metal chlorides in CASMCs.***A*, representative traces of [Ca^2+^]_i_ are changed by NiCl_2_ (100 μM), CoCl_2_ (100 μM), or MnCl_2_ (100 μM) in CASMCs. The cells were preincubated in Hepes-buffered medium (pH 7.4), and at the arrow, the indicated metal chloride was added as described in the Experimental procedures section. *B* and *C*, the dose-dependent effect of acidic pH and NiCl_2_ on [Ca^2+^]_i_ changes. After cells were pre-incubated in Hepes-buffered medium (pH 7.6), they were stimulated for 2 min by the concentrations of NiCl_2_ (1, 3, 10, 30, 100, and 300 μM) or indicated pH (pH 7.6, 7.4, 7.2, 7.0, 6.7, 6.4, and 6.1). Differences between peak and basal values are shown as the means ± SEM (n = 3). Ca^2+^ responses were apparently induced by acidic pH and NiCl_2_ in CASMCs. *D*, the effect of si-OGR1 on the [Ca^2+^]_i_ changes in response to NiCl_2_, acidic pH, and sphingosine-1-phosphate (S1P). After the siRNA-transfected CASMCs were preincubated in Hepes-buffered medium (pH 7.6), the cells were stimulated for 2 min by NiCl_2_ (100 μM), HCl (1 M, final pH 6.4), or S1P (1 μM). Error bars represent the mean ± SEM (n = 3). Comparisons of siRNAs specific to OGR1 (si-OGR1) *versus* control siRNA (si-NS) were assessed using an unpaired Student’s *t*-test. The effect of siRNAs on NiCl_2_ or pH 6.4 action was significant (∗*p* = 0.0108 or ∗∗*p* = 0.0034, respectively); however, that of S1P action was not significant (N.S. *p* = 0.8596). *E*, the effect of YM-2514890 or 2-APB in [Ca^2+^]_i_ in CASMCs. After cells were preincubated in Hepes-buffered medium (pH 7.6), the cells were treated for 2 min with YM-2514890 (100 nM), 2-APB (30 μM), or DMSO as a vehicle and stimulated for 2 min by HCl (1 M, final pH 6.4) or NiCl_2_ (100 μM). Error bars represent the mean ± SEM (n = 3). Comparisons of each test reagent versus DMSO were assessed using a one-way ANOVA, followed by the Tukey test. The effect of YM-2514890 or 2-APB on NiCl_2_ or pH 6.4 was significant (∗∗∗∗*p* < 0.0001 or ∗∗∗*p* < 0.0003. respectively). *F*, the effect of Ca^2+^ chelation with EGTA in [Ca^2+^]_i_ in CASMCs. After cells were preincubated in Hepes-buffered medium (pH 7.6), the cells were treated for 1 min with EGTA (2.5 mM) or water as a vehicle (Veh) and stimulated for 2 min by NiCl_2_ (100 μM) or HCl (1 M, final pH 6.4). Error bars represent the mean ± SEM (n = 3). Comparisons of EGTA and the vehicle were assessed using an unpaired Student’s *t*-test, and the effect of EGTA on NiCl_2_ or pH 6.4 was not significant (N.S. *p* = 0.3098 or N.S. *p* = 0.2585, respectively). 2-APB, 2-aminoethoxydiphenyl borate; CASMC, coronary artery smooth muscle cell; DMSO, dimethyl sulfoxide; HIF, hypoxia-inducible factor; NiCl_2_, nickel chloride; OGR1, ovarian cancer G protein–coupled receptor 1.

We assessed Ca^2+^ mobilization in Fura-2–loaded CASMCs in response to metal chlorides and acidic pH. The intracellular Ca^2+^ concentration ([Ca^2+^]_i_) was measured in cell suspension when gentle stirred with Fura-2–loaded cells. NiCl_2_, CoCl_2_, and MnCl_2_ (100 μM) all triggered [Ca^2+^]_i_ increases (Fig. 5*A*). Due to interference with the detection of intercellular total Fura-2, [Ca^2+^]_i_ changes were reported as 340/380 ratios as described in the Experimental procedures section. Both NiCl_2_ and acidic pH (pH 7.0–6.4) increased [Ca^2+^]_i_ in a dose-dependent manner (Fig. 5, *B* and *C*, respectively). In CASMCs, sphingosine-1-phosphate (S1P) is known to stimulate the S1P receptors-mediated Ca^2+^ signaling pathway (47). OGR1 knockdown specifically reduced NiCl_2_- and pH 6.4-induced [Ca^2+^]_i_ increases but had no effect on S1P-induced responses (Fig. 5*D*). YM254890 (a G_q/11_ inhibitor) and 2-aminoethoxydiphenyl borate (2-APB, an inositol trisphosphate receptor [IP_3_R] blocker) suppressed the [Ca^2+^]_i_ responses (Fig. 5*E*), which remained unchanged after extracellular Ca^2+^ removal (Fig. 5*F*). Thus, Ca^2+^ is mobilized from an intracellular pool, possibly through G_q/11_/PLC activation in CASMCs. These results suggest that metal chlorides stimulate the release of intracellular Ca^2+^ through the OGR1/G_q/11_/PLC signaling pathways.

### Ca^2+^-dependent signaling through PKC, PKD, and CaMKII regulates IL-6 secretion

**Figure 6:**
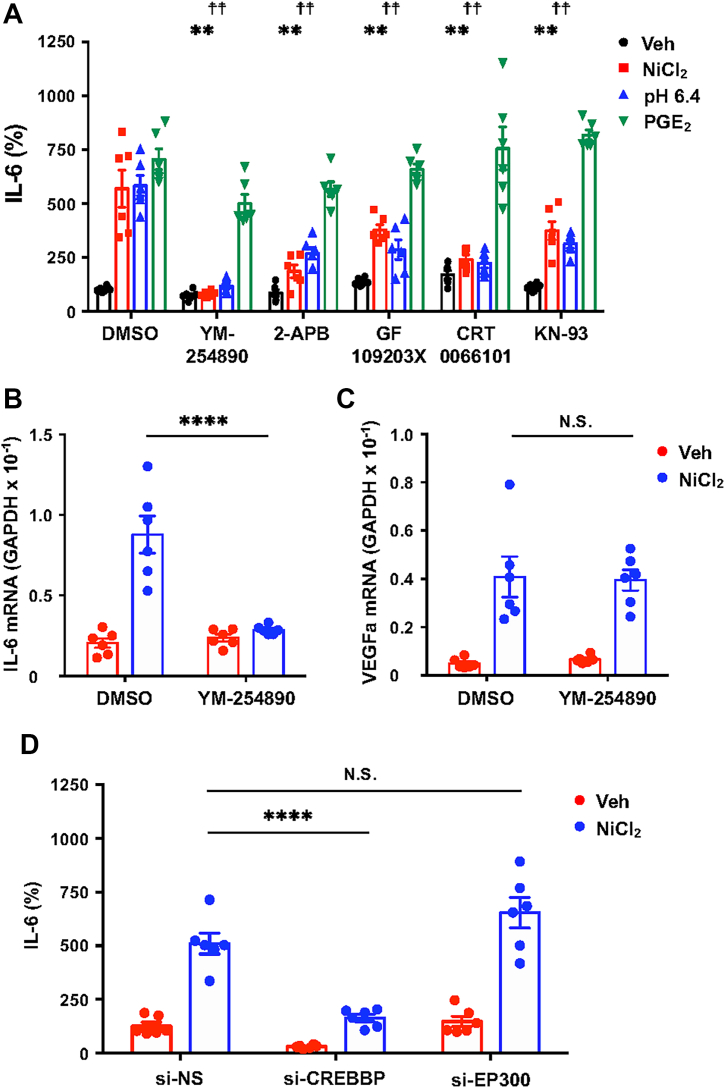
**G_q/11_/Ca^2+^- and CREB-dependent pathways are important for NiCl_2_ and acidic pH signaling in CASMC IL-6 production.***A*, the effect of different inhibitors on the IL-6 production induced by NiCl_2_, acidic pH, and PGE_2_. The cells were pretreated with YM-254890 (100 nM), 2-APB (30 μM), GF109203X (1 μM), CRT0066101 (1 μM), KN-93 (1 μM), or DMSO at 37 °C for 8 h in RPMI-1640-0.1% BSA. The cells were stimulated for 20 h by NiCl_2_ (100 μM), pH 6.4 (adjusted with 1 M HCl), PGE_2_ (1 μM), and the vehicle (Veh) in RPMI-1640-Hepes (pH 7.4)-0.1% BSA containing inhibitors or DMSO. IL-6 proteins were measured by an ELISA method. Results are expressed as percentages of the basal value obtained with DMSO vehicle. Error bars represent the mean ± SEM (n = 6). Comparisons of siRNAs specific to inhibitors *versus* DMSO were assessed using a two-way ANOVA, followed by the Tukey test. The effect of inhibitors on NiCl_2_ or pH 6.4 action was apparent (∗∗*p* < 0.01 *versus* NiCl_2_ with DMSO, ☨☨︎*p* < 0.01 *versus* pH 6.4 with DMSO); however, the effect on PGE_2_ action was slight. YM-254890 and CRT0066101 suppressed the levels to the basal value; however, 2-APB, GF109203X, and KN-93 resulted in partial inhibition. *B* and *C*, the effect of YM-254890 on NiCl_2_-induced mRNA expression for IL-6 and VEGFa. Serum-starved CASMCs were stimulated at 37 °C for 20 h by NiCl_2_ (300 μM) or the vehicle (Veh) in RPMI-1640-Hepes (pH 7.4)-0.1% BSA. The mRNAs for IL6 (B, IL-6) and VEGFA (C, VEGFa) were analyzed by the RT-qPCR method. Results are expressed as relative ratios to GAPDH mRNA expression. Error bars represent the mean ± SEM (n = 6). Comparisons of YM-254890 *versus* DMSO were assessed using a two-way ANOVA, followed by the Tukey test. The effect of YM-254890 on IL-6 expression was significant (∗∗∗∗*p* < 0.0001), but not on VEGFa (N.S. *p* = 0.9969). *D*, the effect of siRNAs specific to CREB transcriptional coactivators on NiCl_2_-induced IL-6 secretion. Serum-starved CASMCs were incubated at 37 °C for 20 h in RPMI-1640-Hepes (pH 7.4)-0.1% BSA with NiCl_2_ (100 μM) or vehicle (Veh). IL-6 proteins were measured by the ELISA method. Results are expressed as percentages of the basal value obtained with the vehicle in si-NS cells. Error bars represent the mean ± SEM (n = 6). Comparisons of siRNAs specific to CREB binding protein (si-CREBBP) or or E1A binding protein p300 (si-EP300) *versus* si-NS were assessed using a two-way ANOVA, followed by the Tukey test. The effect of si-CREBBP on NiCl_2_ action was apparent (∗∗∗∗*p* < 0.0001); however, that of si-EP300 was not significant (N.S. *p* = 0.1126). 2-APB, 2-aminoethoxydiphenyl borate; BSA, bovine serum albumin; CASMC, coronary artery smooth muscle cell; CREB, cAMP-responsive element-binding protein; CREBBP, CREB-binding protein; IL-6, interleukin-6; NiCl_2_, nickel chloride; OGR1, ovarian cancer G protein–coupled receptor 1.

To further define Ca^2+^-dependent pathways downstream of OGR1, we examined the effects of specific inhibitors (Fig. 6*A*). IL-6 secretion induced by NiCl_2_ or pH 6.4 was significantly suppressed by YM254890 and CRT0066101 (a PKD inhibitor) down to basal levels. In contrast, 2-APB, GF109203X (a PKC inhibitor), and KN-93 (a CaMKII inhibitor) produced significant but modest inhibition. None of the inhibitors affected PGE_2_-induced IL-6 production. YM254890 also robustly suppressed IL-6 mRNA expression without affecting VEGFa mRNA (Fig. 6, *B* and *C*), indicating that IL-6 production is predominantly driven by the OGR1/G_q/11_/Ca^2+^ pathway, with PKD playing a central role.

It has been reported that cAMP/CREB signaling is important for the production of IL-6 (17, 18, 19, 20). In addition, the Ca^2+^ signaling pathway with PKD has also been reported to be involved in CREB activation (22, 23). siRNAs targeting CREB-binding protein (CREBBP) and E1A-binding protein p300 (EP300) were employed. Transfection with si-CREBBP and si-EP300 effectively reduced the levels of their respective mRNAs (Fig. S4, *A* and *B*, respectively), and the corresponding proteins were reduced to nearly undetectable levels (Fig. S4*C*). CREBBP knockdown significantly inhibited NiCl_2_-induced IL-6 secretion, whereas EP300 knockdown had no effect (Fig. 6*D*). Thus, IL-6 production is regulated downstream of the OGR1/G_q/11_/Ca^2+^ pathway *via* CREBBP-dependent transcriptional regulation.

### NiCl_2_ and acidic pH stimulate CREB phosphorylation *via* OGR1/G_q/11_ and PKD

**Figure 7:**
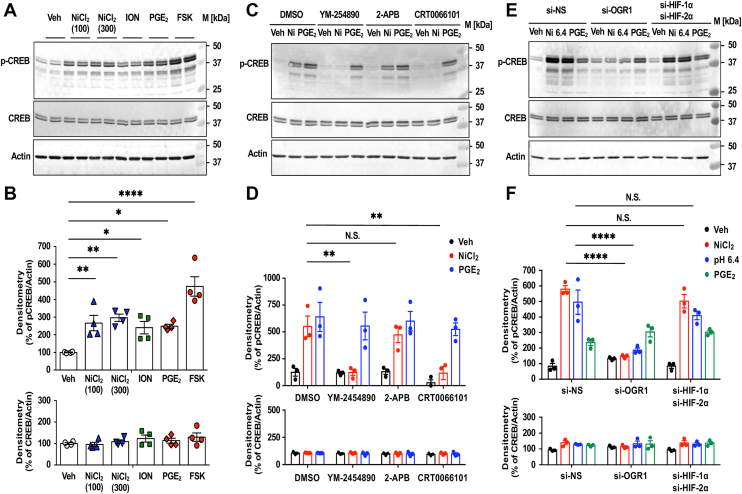
**NiCl_2_ or acidic pH activates CREB through OGR1/G_q/11_ and PKD-dependent pathways.***A* and *B*, the phosphorylation of CREB at Ser133 (p-CREB) is induced by the cAMP pathway or the Ca^2+^ signaling pathway in CASMCs. The CREB activation response to NiCl_2_ (100–300 μM), ION (1 μM), PGE_2_ (1 μM), FSK (10 μM), or the vehicle (Veh) was examined. After serum starvation for 8 h, CASMCs were stimulated at 37 °C for 10 min by the indicated reagents in RPMI-1640-Hepes (pH 7.4)-0.1% BSA. Phospho-CREB and CREB, together with actin contents, were measured in cell lysate by Western blotting as described in the the Experimental procedures section. Gel images are representative results (*A*). The results were also expressed as percentages of each vehicle (*B*). Data are shown as the mean ± SEM of each group (n = 4). Comparisons of each test reagent *versus* the vehicle were assessed using a one-way ANOVA, followed by the Tukey test. The effects of the test reagents compared with the vehicle were significant for NiCl_2_ (100 μM, ∗∗*p* = 0.0106; 300 μM, ∗∗*p* = 0.0027), ION (∗*p* = 0.0336), PGE_2_ (∗*p* = 0.0237), and FSK (∗∗∗∗*p* < 0.0001). *C* and *D*, the effect of different inhibitors of the Ca^2+^ signaling pathway on the CREB activation induced by NiCl_2_ and PGE_2_. After serum starvation for 8 h, CASMCs were pretreated with YM-254890 (100 nM), 2-APB (30 μM), CRT0066101 (1 μM), or DMSO at 37 °C for 30 min in RPMI-1640-0.1% BSA. The cells were stimulated for 10 min by NiCl_2_ (300 μM), PGE_2_ (1 μM), or the vehicle (Veh) in RPMI-1640-Hepes (pH 7.4)-0.1% BSA containing inhibitors or DMSO. Data are shown as the mean ± SEM of each group (n = 3). Gel images are representative results (*C*). The results were also expressed as percentages of each DMSO (*D*). Comparisons of NiCl_2_*versus* DMSO were assessed using a two-way ANOVA, followed by the Tukey test. The effect of YM-25489 or CRT0066101 on NiCl_2_ action was significant (∗∗*p* = 0.0014 or ∗∗*p* = 0.0012, respectively); however, that of 2-APB was not significant (N.S. *p* = 0.8019). *E* and *F*, the effect of siRNAs on the CREB activation by induced NiCl_2_. After serum starvation for 8 h, siRNA-transfected CASMCs were incubated at 37 °C for 10 min in RPMI-1640-Hepes (pH 7.4)-0.1% BSA with NiCl_2_ (300 μM), pH 6.4 (adjusted with 1 M HCl), PGE_2_ (1 μM), or the vehicle (Veh). Error bars represent the mean ± SEM (n = 3). Gel images are representative results (*E*). The results were also expressed as percentages of each vehicle in si-NS cells (*F*). Comparisons of siRNAs specific to OGR1 (si-OGR1) or HIF1A (si-HIF-1α) and EPAS1 (si-HIF-2α) *versus* control siRNA (si-NS) were assessed using a two-way ANOVA, followed by the Tukey test. The effect of si-OGR1 on the NiCl_2_ or pH 6.4 actin was significant (∗∗∗∗*p* < 0.0001); however, that of HIF-1α and si-HIF-2α was not significant (N.S. *p* = 0.1610 and N.S. *p* = 0.1099, respectively). 2-APB, 2-aminoethoxydiphenyl borate; BSA, bovine serum albumin; CASMC, coronary artery smooth muscle cell; CREB, cAMP-responsive element-binding protein; FSK, forskolin; HIF, hypoxia-inducible factor; ION, ionomycin; PGE_2_, prostaglandin E_2_; PKD, protein kinase D; NiCl_2_, nickel chloride; OGR1, ovarian cancer G protein–coupled receptor 1.

We examined the phosphorylation of CREB in response to NiCl_2_ and acidic pH 6.4 (Fig. 7). CREB was activated by NiCl_2_, ION, PGE_2_, and FSK (Fig. 7, *A* and *B*), confirming the involvement of both G_q/11_ and G_s_ pathways. Inhibitors YM254890 and CRT0066101 reduced NiCl_2_-induced CREB phosphorylation, whereas 2-APB did not (Fig. 7, *C* and *D*, respectively), indicating that Ca^2+^ mobilization is not necessary for NiCl_2_-induced CREB activation. OGR1 knockdown abolished CREB phosphorylation induced by NiCl_2_ or acidic pH, whereas HIF-α knockdown had no effect (Fig. 7, *E* and *F*, respectively). These results suggest that CREB is phosphorylated *via* OGR1/G_q/11_/PKD independently of HIFs or G_s_/cAMP signaling in CASMCs.

### PKD2 is a key mediator of OGR1/G_q/11_-induced IL-6 production *via* CREB activation

**Figure 8:**
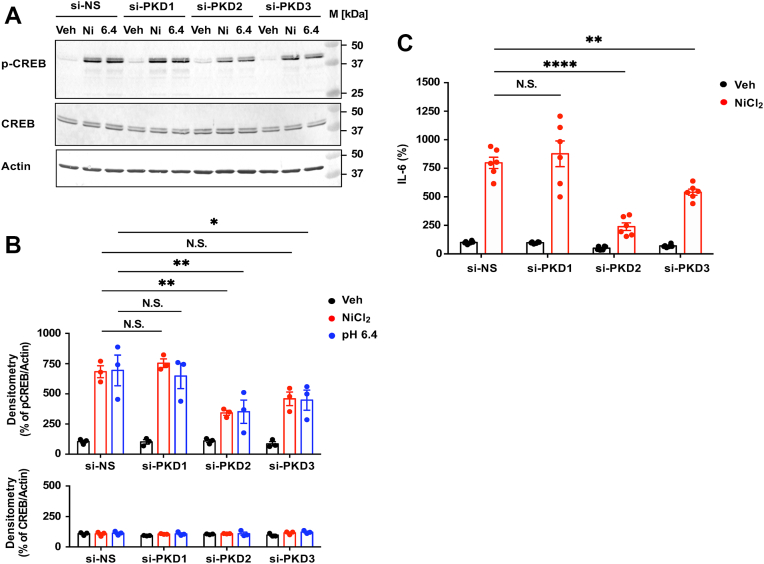
**PKD2- and PKD3-dependent pathways are essentially required for NiCl_2_ or acidic pH-induced IL-6 secretion.***A* and *B*, the CREB activation response to NiCl_2_ and pH 6.4 was examined. After serum starvation for 8 h, CASMCs transfected with siRNAs specific to PKD1-3 were stimulated at 37 °C for 10 min by NiCl_2_ (300 μM), pH 6.4 (adjusted with 1 M HCl), or the vehicle (Veh) in RPMI-1640-Hepes (pH 7.4)-0.1% BSA. The p-CREB and CREB together with the actin contents were measured in cell lysate by Western blotting as described in the Experimental procedures section. Gel images are representative results (*A*). The results were also expressed as percentages of each vehicle in si-NS cells (*B*). Data are shown as the mean ± SEM of each group (n = 3). Comparisons of each test reagent *versus* the vehicle were assessed using a two-way ANOVA, followed by the Tukey test. The phosphorylation of CREB by NiCl_2_ or pH 6.4 apparently suppressed si-PKD2 (∗∗*p* = 0.0033 or ∗∗*p* = 0.0032, respectively) and was slightly inhibited by si-PKD3 (N.S. *p* = 0.0602 or ∗*p* = 0.0361, respectively); however, those of si-PKD1 resulted in a marginal effect (N.S. *p* > 0.7914 or N.S. *p* = 0.9229, respectively). *C*, the effect of siRNAs specific to PKD1–3 on NiCl_2_-induced IL-6 secretion. Serum-starved CASMCs were incubated at 37 °C for 20 h in RPMI-1640-Hepes (pH 7.4)-0.1% BSA with NiCl_2_ (100 μM) or the vehicle (Veh). IL-6 proteins were measured by an ELISA method. Results are expressed as percentages of the basal value obtained with the vehicle in si-NS cells. Error bars represent the mean ± SEM (n = 6). Comparisons of si-PKD1–3 *versus* si-NS were assessed using a two-way ANOVA, followed by the Tukey test. The effect of si-PKD2 or si-PKD3 on NiCl_2_ action was significant (∗∗∗∗*p* < 0.0001 or ∗∗*p* = 0.0010, respectively); however, that of si-PKD1 was not significant (N.S. *p* = 0.4857). BSA, bovine serum albumin; CASMC, coronary artery smooth muscle cell; CREB, cAMP-responsive element-binding protein; HIF, hypoxia-inducible factor; IL-6, interleukin-6; NiCl_2_, nickel chloride; PKD, protein kinase D.

To dissect the role of PKD isoforms in IL-6 production, siRNAs targeting PKD1–3 were used (Fig. S5). Transfection with si-PKD1–3 markedly decreased their respective mRNA levels (Fig. S5, *A*–*C*), and the corresponding proteins were diminished to almost undetectable levels (Fig. S5*D*). PKD2 knockdown substantially reduced CREB phosphorylation in response to NiCl_2_ and pH 6.4, with a minor effect observed for PKD3 and a negligible effect for PKD1 (Fig. 8, *A* and *B*, respectively). Corresponding IL-6 secretion patterns mirrored these results (Fig. 8*C*). Thus, the OGR1/G_q/11_/PKD2/CREB axis plays a predominant role in IL-6 production.

### Acidic pH enhances metal ion–induced IL-6 production *via* OGR1 activation

**Figure 9:**
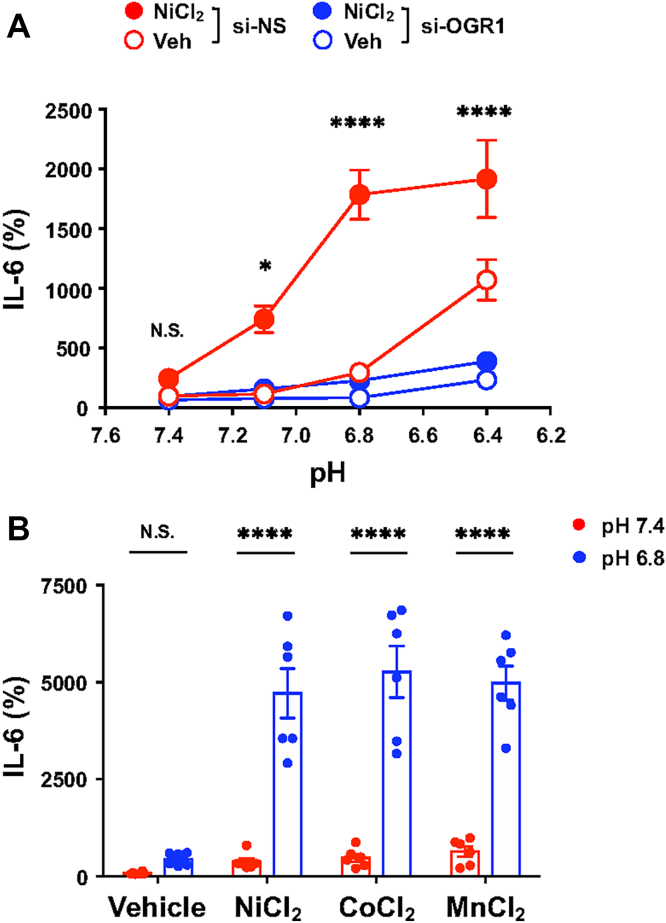
**Subtle extracellular acidification contributes to enhancement for OGR1 signaling in response to metal chlorides.***A*, extracellular pH-dependent IL-6 secretion. After serum starvation for 8 h, CASMCs were incubated at 37 °C for 20 h in RPMI1640-Hepes (pH 7.4, 7.1, 6.8, and 6.4)-0.1% BSA containing NiCl_2_ (10 μM) or the vehicle (Veh). IL-6 proteins were measured by an ELISA method. Results are expressed as percentages of the basal value obtained with the vehicle at pH 7.4. Error bars represent the mean ± SEM (n = 6). Comparisons of NiCl_2_ in si-OGR1 cells *versus* NiCl_2_ in si-NS cells were assessed using a two-way ANOVA, followed by the Tukey test. The effect of si-OGR1 was significant (N.S. *p* = 0.8811, ∗*p* = 0.0103, and ∗∗∗∗*p* < 0.0001, respectively). *B*, the effect of subtle acidification on NiCl_2_-induced IL-6 secretion. Serum-starved CASMCs were incubated at 37 °C for 20 h in RPMI-1640-Hepes (pH 7.4–6.8)-0.1% BSA with metal chlorides (10 μM) or the vehicle. Other experimental procedures are the same as those described in (*A*). Comparisons of each metal chloride at pH 6.8 *versus* that at pH 7.4 were assessed using a two-way ANOVA, followed by the Tukey test. The effect of metal chloride action at pH 6.8 was significant (∗∗∗∗*p* < 0.0001); however, that of the vehicle was not significant (N.S. *p* = 0.9361). BSA, bovine serum albumin; CASMC, coronary artery smooth muscle cell; HIF, hypoxia-inducible factor; IL-6, interleukin-6; OGR1, ovarian cancer G protein–coupled receptor 1; NiCl_2_, nickel chloride.

Ni^2+^ is a component of cardiovascular stents and may contribute to vascular inflammation (3, 14). Prior reports have suggested the allosteric modulation of OGR1 by protons and metal ions (36). Mutational analyses indicate distinct but possibly overlapping domains for metal and proton sensing (35, 36). Therefore, we investigated IL-6 secretion under mildly acidic conditions (pH 6.8). Although pH 6.8 alone slightly increased IL-6 (Fig. 4, *B* and *C*), costimulation with NiCl_2_ (10 μM) significantly amplified this response (Fig. 9*A*). OGR1 knockdown abolished this synergistic effect, which appeared to arise in the proximity of the OGR1 receptor and was also observed at the level of CREB phosphorylation (Figs. 9*A* and S6). Similar responses were also observed with CoCl_2_ or MnCl_2_ (10 μM) at pH 6.8 (Fig. 9*B*), whereas ZnCl_2_ or CuCl_2_ had minimal effects. These results indicate that OGR1-mediated inflammatory responses are enhanced under mildly acidic conditions, potentially reflecting vascular pathophysiological states.

## Discussion

Cytokines and growth factors are known to contribute to vascular inflammation and the subsequent development of vascular disease (48, 49). HIF-α subunits are critical regulators of growth factors and cytokine production in angiogenesis and vascular remodeling (38, 39). In vascular cells, hypoxic conditions influence the expression of various HIF target genes, including IL-6 (2), COX-2 (7), VEGFa (3, 40, 41, 42, 43), and leptin (8, 9). In the present study, we demonstrated that the mRNA expression of these genes exhibits distinct temporal patterns in response to NiCl_2_ (Fig. 1), indicating that mechanisms independent of hypoxia may also be involved. The mRNA-level phenomenon was subsequently accompanied by the production of IL-6 and COX-2 or the release of VEGFa and leptin, which could be corroborated by complementary evidence obtained through Western blotting and ELISA analyses. The induction of HIF-1α and HIF-2α by metal chlorides was confirmed by Western blotting (Fig. 2), although the delayed detection of these responses may reflect the time required for metal ions to traverse the cell membrane *via* transport mechanisms. Notably, the induction of HIF-1α and HIF-2α and downstream signaling events remain unaffected under acidic conditions (Fig. S1*C*).

**Figure 10:**
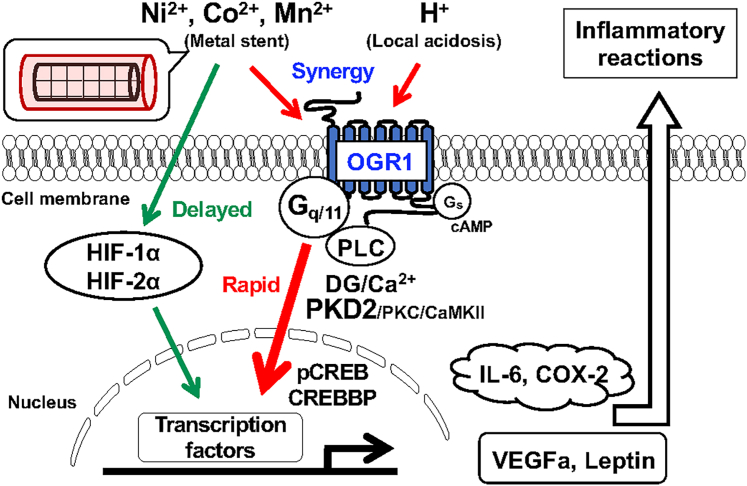
**Inflammatory responses induced by metal ions and acidic pH: cellular responses mediated by OGR1 in addition to HIF-1α and HIF-2α.** The main mechanisms of restenosis after stent implantation are thought to involve neointimal hyperplasia and inflammatory responses. It has been suggested that metal ions released from the stent are involved in these processes through HIF-1α and/or HIF-2α. Furthermore, this study suggests that OGR1, which is a receptor functioning as a sensor for both protons and metal ions, displaying mechanistic synergy under pathological vascular conditions, could trigger signaling pathways such as intracellular Ca^2+^ mobilization and play a role in regulating inflammatory reaction. HIF, hypoxia-inducible factor; OGR1, ovarian cancer G protein–coupled receptor 1.

Our siRNA experiments targeting HIF-1α, HIF-2α, and OGR1 indicated that the expression of VEGFa and leptin is upregulated by NiCl_2_ primarily through HIF-dependent mechanisms, while IL-6 and COX-2 expression appears to be mediated by OGR1 (Figs. 3 and 4, respectively). These findings suggest that metal ion–induced inflammatory responses in CASMCs involve both rapid responses, such as OGR1-mediated IL-6 and COX-2 production, and relatively slower responses mediated by HIFs, such as VEGFa and leptin expression. Together, these pathways may act synergistically to promote inflammatory responses and vascular remodeling, thereby contributing to disease progression. Additionally, local acidification observed in pathological vascular conditions, such as atherosclerosis, may modulate inflammatory signaling *via* OGR1 (Figs. 9 and 10).

Previously, we reported that under acidic pH conditions, OGR1 activation leads to the production of IL-6 and COX-2 in both human aortic and bronchial smooth muscle cells (50, 51). In bronchial smooth muscle cells, we showed that Co^2+^ and Ni^2+^ stimulate IL-6 mRNA expression and protein secretion through OGR1 (44), although the detailed mechanisms remained unclear. In CASMCs, IL-6 production was similarly induced by GPCR agonists such as LPA and PGE_2_, as well as by FSK or ION (Fig. 4), suggesting that this response may be unique to smooth muscle cells. OGR1 expression has been reported in many cell types, with functional OGR1/G_q/11_/Ca^2+^ signaling documented in several of them (27, 29) but notably absent in vascular endothelial cells (29). In the present study, our focus was to define the rapid OGR1-mediated IL-6 response to metal ions or acidic pH in CASMCs. In fact, NiCl_2_, CoCl_2_, and MnCl_2_, as well as acidic pH, appear to induce IL-6 expression through the OGR1/G_q/11_/Ca^2+^ signaling pathway (Figure 6, Figure 7, Figure 8). These metal-induced responses were suppressed by pharmacological inhibitors of G_q/11_, IP_3_R, PKC, PKD, and CaMKII; notably, the G_q/11_ and PKD inhibitors markedly reduced responses down to basal levels (Fig. 6). These effects were accompanied by CREB phosphorylation; notably, CREB phosphorylation was inhibited by a PKD inhibitor but not by an IP_3_R blocker (Fig. 7). Furthermore, siRNA knockdown of PKD2 markedly suppressed both CREB phosphorylation and IL-6 production, while knockdown of PKD3 showed a moderate effect, and that of PKD1 had only a marginal impact (Fig. 8). Thus, NiCl_2_-induced IL-6 production is mediated through the OGR1/G_q/11_/Ca^2+^ pathway, with downstream signaling predominantly involving PKD (particularly PKD2)/CREB, and may be at least in part modulated by PKC and CaMKII.

The downstream signaling also appears to involve the CREB–CREBBP axis (Fig. 6). Although OGR1 has been reported to activate both PLC and adenylate cyclase in receptor-overexpressing CHO cells and COS7 cells under mildly acidic conditions around pH 6.5 (28, 46), we did not observe intracellular cAMP accumulation in CASMCs in response to either NiCl_2_ or acidic pH. This is in contrast to the robust response elicited by PGE_2_ (Fig. 4). Additionally, inhibitors of PKA (H-89) or adenylate cyclase (MDL-12,330A and SQ22,536) had little effect on NiCl_2_- or acid-induced IL-6 production. These results indicate that the OGR1/G_s_/cAMP signaling axis is largely nonfunctional in CASMCs.

In contrast to our previous reports regarding bronchial smooth muscle cells (44), the metal-induced phosphorylation of extracellular signal-regulated kinase 1/2 and p38 mitogen-activated protein kinase in CASMCs was barely detectable. To fully elucidate the mechanisms underlying vascular inflammation, it is essential to consider receptor-mediated signaling not only in smooth muscle cells but also in endothelial and immune cells of hematopoietic origin. Future studies should incorporate vascular tissue cultures or coculture systems that include these diverse cell populations. Moreover, the roles of mitogen-activated protein kinase and NF-κB in coordinating intercellular responses merit further investigation.

Consistent with our previous reports on bronchial smooth muscle cells (44), NiCl_2_, CoCl_2_, and MnCl_2_ induced increases in [Ca^2+^]_i_ *via* OGR1 in CASMCs (Fig. 5). These responses were abolished by inhibitors of G_q/11_-protein or IP_3_R (Fig. 5*E*), with siRNA against OGR1 showing an inhibitory effect slightly greater on NiCl_2_-induced [Ca^2+^]_i_ elevation than that induced by acidic pH (Fig. 5, *D* and *E*, respectively). This difference might reflect the modulating contribution of intracellular acidification or the involvement of additional proton-sensing ion channels or receptors that do not respond to metal ions. Nonetheless, our data support the notion that OGR1 can sense metal chlorides and trigger [Ca^2+^]_i_ signaling in CASMCs.

The concentration of metal ions used in this study reached 300 μM, a level sufficient for attaining a plateau in the dose-response curves for NiCl_2_-induced IL-6 production. At concentrations ≥ 300 μM, CoCl_2_ caused precipitate-like formations around cells after 20 h. Fe^2+^ appeared to exert cytotoxicity through reactive oxygen species, as evidenced by its failure to induce sustained responses such as IL-6 mRNA expression or protein secretion. In contrast, rapid [Ca^2+^]_i_ responses were induced by NiCl_2_, CoCl_2_, MnCl_2_, and FeCl_2_, with FeCl_2_ showing a response profile similar to that of NiCl_2_. FeCl_3_, ZnCl_2_, and CuCl_2_ had only minor effects. The physiological roles of Fe^2+^ in oxygen transport and metabolism are tightly regulated—typically *via* protein binding—to prevent toxicity. It remains an unanswered question whether Fe^2+^ is aberrantly exposed under pathological conditions or whether protein-bound Fe^2+^ can engage in OGR1-mediated signaling.

Previous studies have reported the allosteric modulation of OGR1 by metal ions and protons in receptor-overexpressing HEK293T cells (36), suggesting that OGR1 may be capable of detecting subtle microenvironment shifts under mildly acidic conditions. In this study, OGR1-mediated IL-6 production was triggered under conditions exceeding 10 μM NiCl_2_ or pH levels below 6.8 (Fig. 4, *B* and *C*, respectively). This supports the hypothesis that mildly acidic environments enhance OGR1's sensitivity to trace metal ions (Figs. 9 and S6), promoting inflammatory responses relevant to vascular pathology. Mutational analysis has shown that metal ion sensing involves OGR1's extracellular domain, which may not fully overlap with the proton-sensing region (35, 36). Thus, understanding OGR1’s role in metal ion–induced inflammation may lead to new avenues for therapeutic intervention.

Systemic Ni^2+^ concentrations in humans have been reported to be 0.47 ng/ml (∼8 nM), with the implantation of an Amplatzer device resulting in a peak approximately four times the baseline after 1 month (52). Similarly, in minipigs implanted with a nitinol stent, the serum Ni^2+^ concentration remained around 1 ppb (∼17 nM), with no significant increase from baseline over 180 days (53). These findings suggest that the concentrations of metal ions entering systemic circulation are relatively low. However, local concentrations at the stent site remain unknown and may be substantially higher, particularly under acidic conditions. Although many *in vitro* studies have employed 0.5 to 1.5 mM NiCl_2_ to induce HIF (2, 3), our finding that IL-6 induction occurs at ≤ 10 μM NiCl_2_ under acidic pH conditions is considered clinically relevant.

In summary, we investigated the intracellular pathways involved in OGR1-mediated IL-6 production in CASMCs and compared them with HIF-mediated hypoxia-like responses induced by metal ions. We conclude that IL-6 production is at least partially mediated through the OGR1/G_q/11_/Ca^2+^ signaling axis, particularly involving PKD2 and CREB. Given that neointimal hyperplasia and inflammation are key drivers of restenosis after stent implantation, our findings suggest that both rapid OGR1-mediated and delayed HIF-mediated responses to metal ions may jointly regulate vascular inflammation. Further studies into proton-sensing GPCRs in vascular inflammation may help to identify novel therapeutic targets for vessel injury, particularly in the context of stent-associated complications.

## Experimental procedures

### Materials

Human CASMCs (catalog No. CC-2583) originated from nondiseased individuals were purchased from Lonza; nickel (II) chloride hexahydrate (NiCl_2_), cobalt (II) chloride hexahydrate (CoCl_2_), manganese (II) chloride tetrahydrate (MnCl_2_), iron (II) chloride tetrahydrate (FeCl_2_), iron (III) chloride hexahydrate (FeCl_3_), zinc chloride (ZnCl_2_), copper (II) chloride dihydrate (CuCl_2_), PGE_2_, GF 109203X, YM-254890, RPMI-1640 medium (189-02025), RPMI-1640-Hepes medium (189-02145), and penicillin-streptomycin solution (x100) were from FUJIFILM Wako Pure Chemical; anti-actin antibody (A5060), basic fibroblast growth factor (recombinant human), epidermal growth factor (recombinant human), insulin (recombinant human), 1-oleoyl-sn-glycero-3-phosphate (LPA) and S1P, FSK, PMA, ION, 2-APB, 3-isobutyl-1-methylxanthine, N-(2-[p-bromocinnamylamino]ethyl)-5-isoquinolinesulfonmide (H89), MDL-12,330A, SQ22,536, and EGTA were from Sigma-Aldrich; CRT0066101, KN-92, and cAMP ELISA Kit were from Cayman Chemical Co; human IL-6 ELISA (88-7066), human VEGF ELISA Kit (KHG0112), human Leptin ELISA Kit (KAC2281), and BCA Protein Assay were from Thermo Fisher Scientific; Fura-2/acetoxymethyl ester was from Dojindo; anti-CREB antibody (48H2), anti-phospho-CREB (Ser133) antibody (87G3), anti-CREB binding protein antibody (D6C5), E1A binding protein p300 (EP300, D2X6N), anti-HIF-1α antibody (D2U3T), anti-HIF-2α antibody (D6T8V), anti-protein kinase D1 antibody (PKD, D4J1N), anti-protein kinase D2 antibody (PKD2, D1A7), and anti-protein kinase D3 antibody (PKD3, D57E6) were from Cell Signaling Technology; fatty acid–free bovine serum albumin (BSA, Fraction V) was from Calbiochem-Novabiochem Co; nonsilencing control siRNA (si-NS, D-001810-10), and siRNAs specific to human GPR68 (si-OGR1, L-005591-00), HIF1A (si-HIF-1α, L-004018-00), CREB binding protein (si-CREBBP, L-003477-00), E1A binding protein p300 (si-EP300, L-003486-00), protein kinase D1 (si-PKD1, L-005028-00), protein kinase D2 (si-PKD2, L-004197-00), and protein kinase D3 (si-PKD3, L-005029-00) were from Dharmacon; control siRNA-A (si-NS2, sc-37007) and siRNA specific to human EPAS1 (HIF-2α, si-35316) were from Santa Cruz Biotechnology; Lipofectamine RNAiMAX Reagent was from Invitrogen; and quantitative real-time PCR (RT-qPCR) probes specific for human GPR68 (OGR1, Hs00268858), GPR65 (TDAG8, Hs01087326), GPR4 (Hs00947870), GPR132 (G2A, Hs00203431), IL6 (IL-6, Hs00174131), COX2 (COX-2, Hs01573469), VEGFA (VEGFa, Hs00173626), LEP (leptin, Hs00174877), HIF1A (HIF-1α, Hs 00153153), EPAS1 (HIF-2α, Hs01026149), CREBBP (Hs00932878), EP300 (Hs00914212), PKD1 (Hs00177037), PKD2 (Hs00212828), PKD3 (Hs01019859), and GAPDH (4352934E) were from Applied Biosystems. The sources of all other reagents were the same as described previously (32, 37, 44, 54, 55, 56).

### Cell culture and the evaluation of cellular activities

Human CASMCs were cultured in RPMI-1640 medium supplemented with 10% (v/v) fetal bovine serum, 1 ng/ml human epidermal growth factor, 4 ng/ml human fibroblast growth factor-2, 5 μg/ml insulin, and penicillin-streptomycin solution (x1) at 37 °C in a humidified air/CO_2_ (19:1) atmosphere. CASMCs were plated on rat-tail collagen (400 μg/ml)-coated 10 cm dishes and were maintained until confluent. Cells with 7 to 10 passages were used for experiments. We confirmed that these cells were positive to staining with α-actin, a smooth muscle cell marker, as described previously (54). To assess the metal ion-stimulated CASMC responses, the cells were plated on collagen-coated 10 cm dishes for the evaluation of [Ca^2+^]_i_ change; on 6 cm dishes for the analysis of gene expressions of interest, CREB phosphorylation, and HIF protein induction; on 12-well plates for the confirmation of cAMP accumulation; and on 24-well plates for the estimation of IL-6 production. Plates were maintained for 72 h at 37 °C in the growth medium. Where indicated, siRNAs were transfected to cells within 24 h after plating subculture as described below. The culture medium was changed to fresh RPMI-1640 containing 0.1% BSA (RPMI-1640-0.1% BSA) to make them quiescent for 8 h. The cells were then stimulated by the indicated concentration of metal ions in RPMI-1640-Hepes medium containing 0.1% BSA (RPMI-1640-Hepes (pH 7.4)-0.1% BSA). The effect of metal chlorides was compared with those of various chemical compounds, such as LPA, S1P, PGE_2_, FSK, ION, and PMA under an appropriate pH with 1 M HCl. The RPMI-1640-Hepes (pH7.4)-0.1% BSA (including acidic pH) was equilibrated for 24 h before experiments at 37 °C in a humidified air/CO_2_ (19:1) atmosphere.

### Transfection of siRNA

The siRNA was transfected into CASMCs using Lipofectamine RNAiMAX reagent as described previously (55). In brief, the cells were harvested with 0.05% trypsin–EDTA and washed with RPMI-1640 medium with 10% fetal bovine serum. The cell suspension (approximately ∼10^7^ cells in 9 ml) was mixed with siRNA solution (60 pmol siRNA and 10 μl of RNAiMAX reagent in 1 ml Opti-MEMI) and plated on 24-well plates, 6 cm dishes, and 10 cm dishes in the same medium. After incubation with siRNAs for 24 h, the medium was changed to the growth medium, and the cells were cultured for 48 h. The growth medium was changed to fresh RPMI-1640 containing 0.1% BSA to make them quiescent for experiments of HIF-protein induction, IL-6 production, RNA analysis, CREB phosphorylation, and [Ca^2+^]_i_ change. The experiments were carried out within 96 h of transfection. To verify the target specificity of si-RNAs toward their mRNA, total RNA was prepared from adherent CASMCs with si-RNAs on 6 cm dishes and subsequent quantitative analysis of mRNA expression was performed as described in the RNA analysis. CASMCs were transfected with si-RNAs on 6 cm dishes, and subsequent Western blotting was performed to assess the specificity of si-RNAs for respective protein expression as described for the Western blot analysis.

### RNA analysis

Serum-starved CASMCs on 6 cm dishes were stimulated by metal ions in RPMI-1640-Hepes (pH 7.4)-0.1% BSA under an appropriate pH with 1 M HCl. Total RNA was prepared from adherent cells in accordance with the manufacturer's instructions for RNAiso Plus (TAKARA BIO Inc). RT-qPCR was performed using TaqMan hydrolysis probes (Applied Biosystems) as described previously (56, 57). The total RNA (5 μg) was treated with DNase I to remove possible traces of genomic DNA and subjected to RT-qPCR. The thermal cycling conditions were as follows: 2 min at 50 °C, 10 min at 95 °C, 45 cycles of 15 s at 95 °C, and 1 min at 60 °C. The expression level of the target mRNA was normalized to the relative ratio of the expression of GAPDH mRNA.

### Western blot analysis

CASMCs in 6 cm dishes were washed twice with ice-cold PBS and harvested from the dishes with a rubber policeman by adding a lysis buffer composed of PBS, 1% IGEPAL CA-630 (Sigma-Aldrich), 0.5% sodium deoxycholate, 0.1% SDS, 1 mM EDTA, and 1% proteinase inhibitor cocktail (Sigma-Aldrich). The lysate was incubated for 30 min on ice and centrifuged at 14,000*g* for 20 min. The protein concentration of the supernatant was determined with a BCA Protein Assay, and equal amounts (15–30 μg) were loaded in each lane in all electrophoresis batches. The recovered lysates were subjected to 10 to 12.5% SDS-PAGE and analyzed by Western blotting with primary antibodies. The membranes were then incubated with a second antibody conjugated with alkaline phosphatase, the blots were visualized using the NBT/BCIP system, and the scanned bands were quantified by ImageJ (http://imagej.nih.gov/ij/index.html) and Quantification of Gel Bands by an ImageJ Macro, Band/Peak Quantification Tool (https://dx.doi.org/10.17504/protocols.io.7vghn3w). The expression level of the target protein was normalized to the relative ratio of actin (A5060, Sigma-Aldrich) as described previously (56).

### Measurement of IL-6, VEGFa, and leptin in the medium using ELISA

The cultured CASMCs on 24-well plates were serum deprived for 8 h in RPMI-1640 containing 0.1% BSA. The serum-starved cells were stimulated by replacing the medium with RPMI-1640-Hepes (pH7.4)-0.1% BSA containing any of the reagents of interest including acidic pH or the vehicle. The RPMI-1640-Hepes media from 24-well plates after incubation for 20 h were collected by centrifugation at 14,000*g* for 1 min. The pH in each sample was adjusted to around 7.4 by the addition of 1 M NaOH and stored at −80 °C until the cytokine content was evaluated. To investigate the effects of specific inhibitors on IL-6 secretion, inhibitors were added to the serum-starvation medium and the stimulation medium. A commercially available ELISA kit (human IL-6/88-7066, human VEGF/KHG0112, and human leptin/KAC2281, Thermo Fisher Scientific) was used to determine the IL-6, VEGFa, and leptin concentrations in accordance with the instruction manual. The adherent cells were washed twice with PBS, followed by treatment with 5% trichloroacetic acid. The trichloroacetic acid-insoluble material was solubilized with a mixture containing 2% Na_2_CO_3_, 0.1% SDS, and 0.1 M NaOH. The protein concentrations of protein extracts were determined with a BCA Protein Assay. The results are shown as the ng/mg protein of adherent CASMCs. The data of extracellular IL-6 content are also expressed as percentages of the basal value obtained with the vehicle.

### Measurement of COX-2, HIF-1α, and HIF-2α proteins

Serum-starved CASMCs on 6 cm dishes were incubated at 37 °C in the RPMI-1640-Hepes (pH7.4)-0.1% BSA together with test substances under an appropriate pH for the indicated times. After incubation, the cells were washed twice with ice-cold PBS and harvested from the dishes with a rubber policeman by adding the lysis buffer as described for the Western blot analysis. The recovered lysate was subjected to 10% SDS-PAGE and analyzed by Western blotting with anti-HIF-1α antibody (D2U3T, Cell Signaling Technology), anti-HIF-2α antibody (D6T8V, Cell Signaling Technology), anti-COX-2-antibody, and anti-actin antibody (A5060, Sigma-Aldrich). Anti-COX-2 antibody was prepared against a synthetic COOH-terminal peptide (ASSSRSGLDDINPT) of COX-2 as described previously (50).

### Estimation of CREB activation

Anti-phosphorylated antibodies against CREB (Ser133) antibody (87G3, Cell Signaling Technology), together with anti-CREB antibody (48H2, Cell Signaling Technology), were used to estimate the CREB activation. The serum-starved CASMCs on 6 cm dishes were stimulated for 10 min at 37 °C by replacing the medium with RPMI-1640-Hepes (pH 7.4)-0.1% BSA containing any of the reagents of interest, including acidic pH or the vehicle. Reactions were terminated by washing twice with ice-cold PBS containing a 1% phosphatase inhibitor cocktail (Sigma-Aldrich) and adding the lysis buffer with a 1% phosphatase inhibitor cocktail and a 1% proteinase inhibitor cocktail. Cytosol fractions were prepared and subjected to 12.5% SDS-PAGE and analyzed by Western blotting with primary antibodies. The scanned bands were quantified by Quantification of Gel Bands by an ImageJ Macro, Band/Peak Quantification Tool, and the phosphorylation level of the target protein was normalized to the relative ratio of actin as described above.

### Measurement of [Ca^2+^]_i_ in Fura-2–loaded cells

Serum-starved CASMCs on 10 cm dishes were gently harvested with PBS containing 0.05% trypsin–EDTA. After a 20 min incubation of the cells with 1 μM Fura-2/acetoxymethyl ester at 37 °C in Ham’s F-10 medium containing 0.1% BSA, the cells were washed two times with ice-cold Hepes-buffered medium composed of 20 mM Hepes (pH 7.4 or 7.6), 134 mM NaCl, 4.7 mM KCl, 1.2 mM KH_2_PO_4_, 1.2 mM MgSO_4_, 2 mM CaCl_2_, 2.5 mM NaHCO_3_, 5 mM glucose, and 0.1% BSA. They were finally suspended in the same medium. Fura-2-loaded cells were warmed for 3 min at 37 °C, and then the [Ca^2+^]_i_ change was monitored at intensities of 540 nm fluorescence obtained by the two excitations (340 nm and 380 nm), which were monitored by a CAF-110 fluorometer (JASCO). The cell suspensions were stimulated for 2 min by the addition of test reagents. Where indicated, an appropriate pH was adjusted with 1 M HCl. As shown in Figure 5, extracellularly added Ni^2+^, Co^2+^, and Mn^2+^ were not rapidly taken up, allowing OGR1-mediated intracellular Ca^2+^ mobilization to be monitored with Fura-2 (340/380 nm ratio). Co^2+^ and Mn^2+^ interfered with the Ca^2+^–Fura-2 interaction, preventing the quantitative assessment of Ca^2+^ concentration, whereas Ni^2+^ (∼100 μM) had little effect and thus permitted reliable measurement of Ca^2+^ mobilization. The net change in [Ca^2+^]_i_ at approximately 20 s was calculated (Δ[Ca^2+^]_i_ (nM) = peak value − baseline value). Other experimental conditions were the same as those previously described (37).

### Intracellular cAMP accumulation

Serum-starved CASMCs on 12-well plates were washed once and preincubated for 10 min at 37 °C in Hepes-buffered medium (pH 7.4) containing 0.1% BSA. The medium was then replaced with the same medium containing test reagents and 0.5 mM 3-isobutyl-1-methylxanthine under an appropriate pH. After a 30 min incubation at 37 °C, the reaction was terminated by the addition of 0.1 M HCl. cAMP in the acid fraction (0.2 ml) was diluted with an ELISA buffer and measured according to the instruction manual of the cAMP ELISA Kit, as described previously (32). The protein concentrations of protein extracts from adherent cells were determined with the BCA Protein Assay as described above. cAMP accumulation is expressed as pmol/mg protein of the adherent cells.

### Statistical analysis

GraphPad Prism 6 (https://www.graphpad.com) was used for statistical calculations. The results of multiple observations are presented as the mean ± SEM or as representative results from more than three different experiments. A Student’s *t*-test for two-group comparisons and a one-way ANOVA, followed by the Tukey test, were used to determine differences between the control and experimental groups, and a two-way ANOVA, followed by the Tukey test, was used to determine the differences between multiple-group comparisons: values were considered significant at *p* < 0.05.

## Data availability

Data are available in the main text or the Supporting information.

## Supporting information

This article contains Supporting information.

## Conflict of interest

The authors declare that they have no conflicts of interest with the contents of this article.
