# Supplementary material for: The proton-sensing OGR1 receptor and hypoxia-inducible factors promote metal ion–induced inflammatory responses in coronary artery smooth muscle cells
Source: J Biol Chem. 2025 Oct 22;301(12):110842. doi: 10.1016/j.jbc.2025.110842 (PMC12661440; doi:10.1016/j.jbc.2025.110842)
Supplement: Supporting Figures [file mmc1.pdf]

Supporting Information

The proton-sensing OGR1 receptor and hypoxia-inducible factors promote metal ion-induced inflammatory responses in coronary artery smooth muscle cells

Koichi Sato[1,2,*], Chihiro Mogi[3], Haruka Aoki-Saito[4], Tamotsu Ishizuka[5], Jun Shirakawa[2], Hideaki Tomura[6], and Dong-Soon Im[7]

[1]Laboratory of Signal Transduction, Institute for Molecular and Cellular Regulation, Gunma University, Maebashi 371-8512, Japan. [2]Laboratory of Diabetes and Metabolic Disorders, Institute for Molecular and Cellular Regulation, Gunma University, Maebashi 371-8512, Japan. [3]Laboratory of Mucosal Ecosystem Design, Institute for Molecular and Cellular Regulation, Gunma University, Maebashi 371-8512, Japan.[4]Department of Respiratory Medicine, Gunma University Graduate School of Medicine, Maebashi 371-8511, Japan. [5]Department of Respiratory Medicine, Faculty of Medical Sciences, University of Fukui, 23-3 Matsuoka-Shimoaizuki, Eiheiji, Fukui 910-1193, Japan. [6]Laboratory of Cell Signaling Regulation, Department of Life Sciences, School of Agriculture, Meiji University, Kawasaki 214-8571, Japan. [7]College of Pharmacy, Kyung Hee University, Seoul 02447, Republic of Korea.

*Correspondence and requests for materials should be addressed to K.S. (E-mail: kosato@gunma-u.ac.jp; Tel: 81-27-220-8853; ORCID 0000-0001-7694-4014)

List of Figures included:

1. Figure S1
2. Figure S2
3. Figure S3
4. Figure S4
5. Figure S5
6. Figure S6

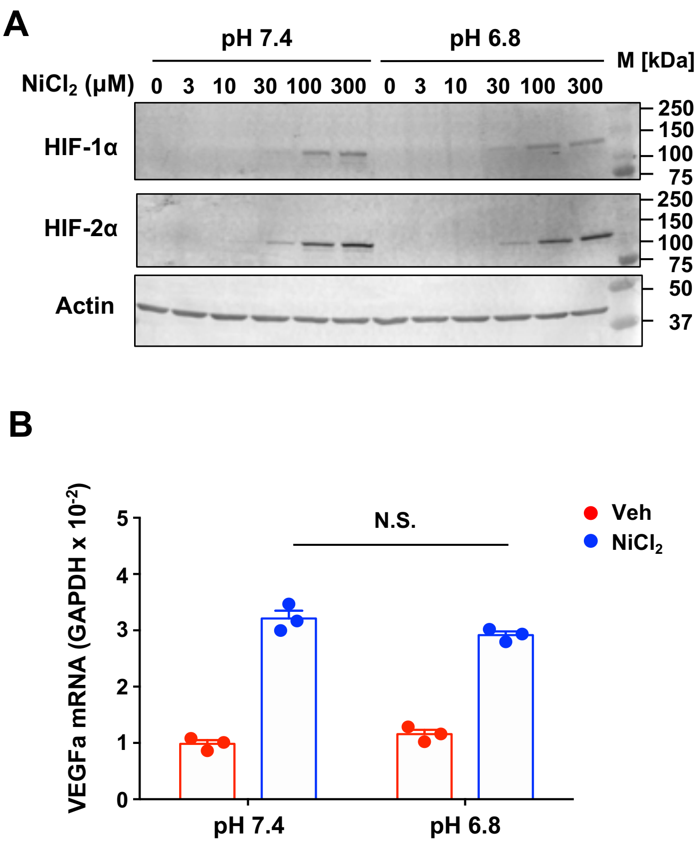

Figure S1. Extracellular acidification had a marginal effect on the induction of HIF signaling by $NiCl_2$ in CASMCs.

(A) Dose-dependent effect of acidic pH on the $NiCl_2$-induced HIF-1α and HIF-2α. After serum starvation for 8 hrs, the cells were incubated at 37°C for 20 hrs in RPMI-1640-HEPES (pH 7.4-6.8)-0.1% BSA with the indicated concentration of $NiCl_2$ or the vehicle. The HIF-1α and HIF-2α contents were measured in cell lysate by Western blotting as described in the Experimental procedures section. Gel images are representative results of three separate experiments. (B) The effect of siRNAs on $NiCl_2$-induced gene expression. Serum-starved CASMCs were stimulated at 37°C for 20 hrs by $NiCl_2$ (300 μM) or the vehicle (Veh) in RPMI-1640-HEPES (pH 7.4-6.8)-0.1% BSA. The expression of VEGFa was analyzed by the RT-qPCR method. Results are expressed as relative ratios to GAPDH mRNA expression. Error bars represent the mean ± SEM (n = 3). Comparisons of $NiCl_2$ vs. the vehicle were assessed using a two-way ANOVA, followed by the Tukey test. The effect of acid pH on the $NiCl_2$ action was not significant (N.S. $p = 0.1800$).

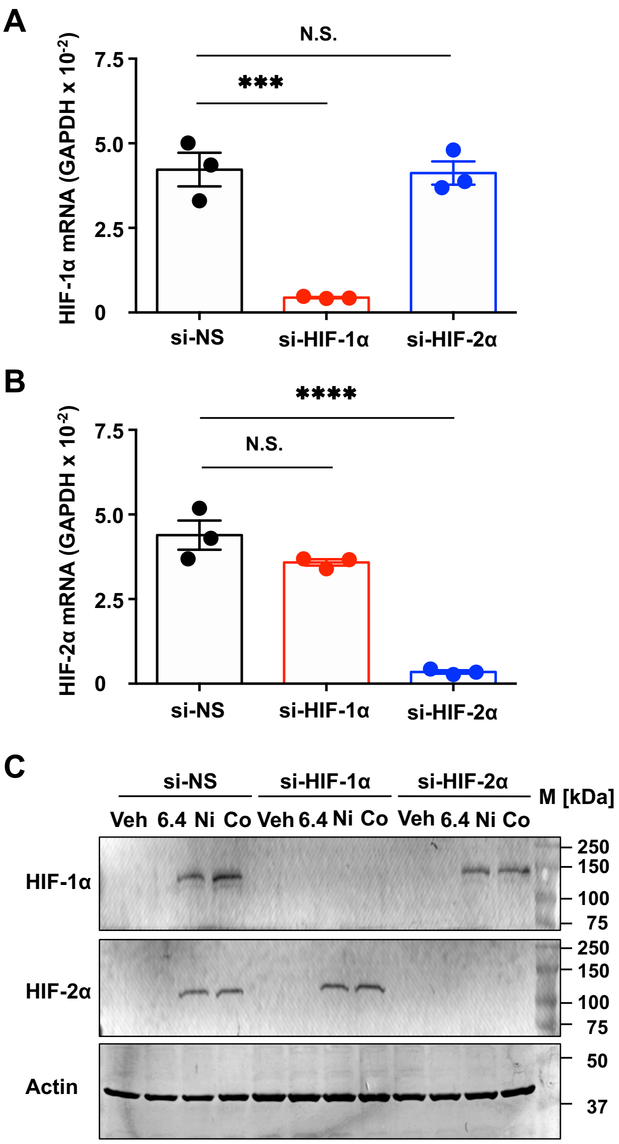

Figure S2. Expression of HIF-1α and HIF-2α in CASMCs and their siRNA specificity. (A, B) To verify the target specificity of si-HIF-1α (HIF1A) and si-HIF-2α (EPAS1) toward HIF-1 and HIF-2α transcripts, CASMCs were subjected to transfection with si-HIF-1, si-HIF-2α or a non-specific control siRNA (si-NS), and the subsequent quantitative analysis of mRNA expression was performed. Results are expressed as relative ratios to GAPDH mRNA expression. Error bars represent the mean ± SEM (n = 3). Comparisons of si-HIF-1α or si-HIF-2α vs. si-NS were assessed using a one-way ANOVA, followed by the Tukey test. The effect of si-HIF-1α or si-HIF-2α was significantly different than that of si-NS. The mRNA expression patterns for HIF-1α (A, ***$p = 0.0005$; N.S. $p = 0.9703$) and HIF-2α (B, N.S. $p = 0.1167$; ****$p < 0.0001$) show the specific downregulation of the respective mRNA of HIF-1α and HIF-2α under the condition. (C) The effect of si-HIF-1α or si-HIF-2α on the induction of HIF-proteins by metal chlorides in CASMCs. After the transfection of si-HIF-1α, si-HIF-2α, or si-NS, CASMCs were stimulated at 37°C for 20 hrs by $NiCl_2$ (300 μM), $CoCl_2$ (300 μM), pH 6.4 (adjusted with 1 M HCl), or the vehicle (Veh) in RPMI-1640-HEPES (pH 7.4)-0.1% BSA. The expression of HIF proteins was analyzed by Western blotting. Gel images are representative results of three separate experiments. Transfection with si-HIF-1α or si-HIF-2α under the experimental condition markedly attenuated the expression of the respective HIF proteins.

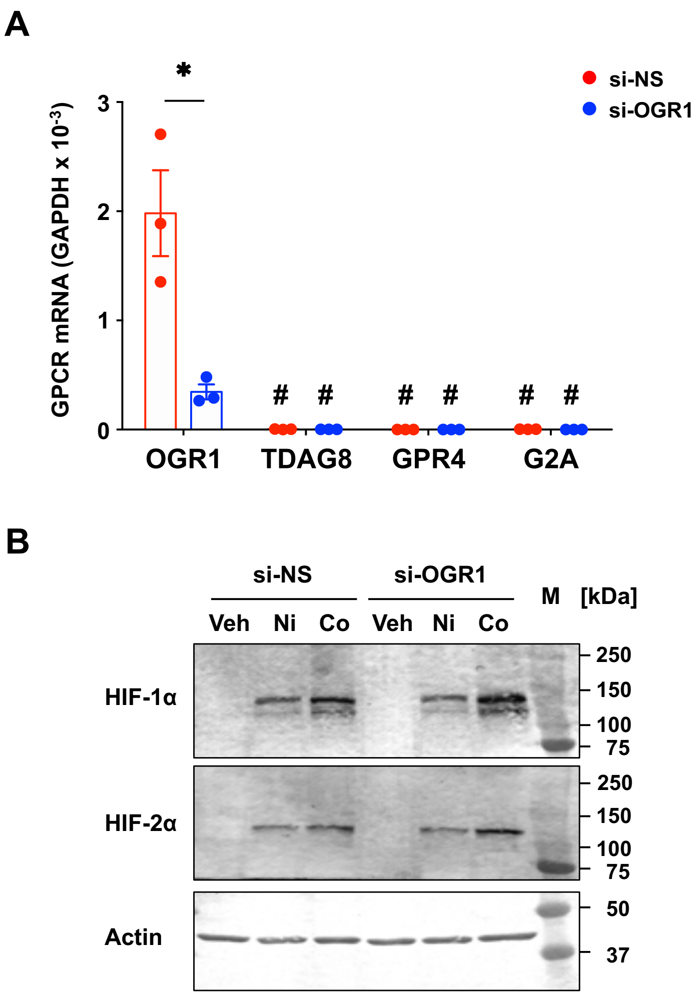

Figure S3. Effect of si-OGR1 on the expression of OGR1-family GPCRs and the accumulation of HIF proteins in CASMCs.

(A) To assess the specificity of si-OGR1 for OGR1 expression, CASMCs were transfected with si-OGR1 or si-NS, followed by the analysis of mRNA expression. The expression of OGR1-family receptor mRNAs in CASMCs was estimated by RT-qPCR. #TDAG8, GPR4, and G2A mRNAs were undetectable (≤ GAPDH x 10^{-6}). Comparisons of si-OGR1 vs. si-NS were assessed using a two-way ANOVA, followed by the Tukey test. The OGR1 expression pattern shows the specific downregulation of the respective mRNA ($*p = 0.0149$). (B) The effect of si-OGR1 on the HIF-1α and HIF-2α induction by metal chlorides in CASMCs. After the transfection of si-OGR1 or si-NS, CASMCs were stimulated at 37°C for 20 hrs by $NiCl_2$ (300 µM) and $CoCl_2$ (300 µM), or the vehicle (Veh) in RPMI-1640-HEPES (pH 7.4)-0.1% BSA. The expression of HIF proteins was analyzed by Western blotting. Gel images are representative results of two separate experiments. The transfection of si-OGR1 did not alter the induction of HIF-1α and HIF-2α stimulated by $NiCl_2$ and $CoCl_2$ in CASMCs.

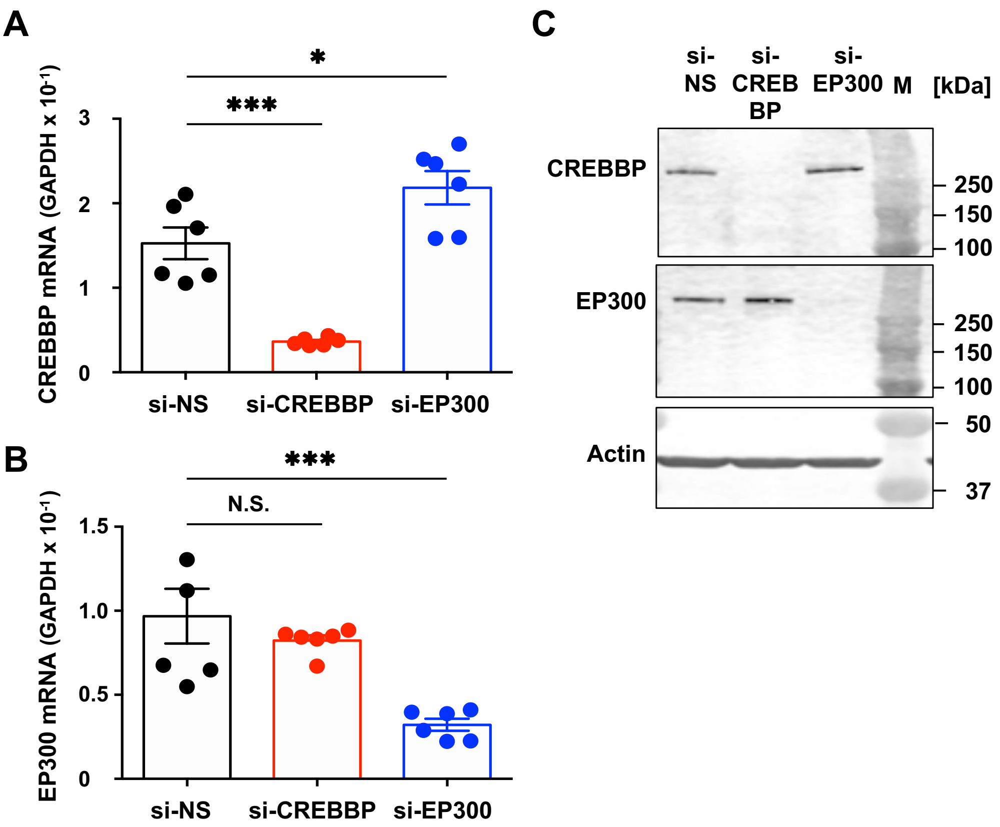

Figure S4. Expression of CREB transcriptional coactivators (CREBBP and EP300) in CASMCs and their siRNA specificity.

(A, B) To verify the target specificity of si-CREBBP and si-EP300 toward CREBBP and EP300 transcripts, CASMCs were subjected to transfection with either si-CREBBP, si-EP300, or si-NS, and the subsequent quantitative analysis of mRNA expression was performed. The expressions of CREBBP and EP300 mRNA were measured by the RT-qPCR method. Results are expressed as relative ratios to GAPDH mRNA expression. Error bars represent the mean ± SEM (n = 6). Comparisons of si-CREBBP or si-EP300 vs. si-NS were assessed using a one-way ANOVA, followed by the Tukey test. The effect of si-CREBBP or si-EP300 was significantly different than that of si-NS. The mRNA expression pattern for CREBBP (A, ***$p = 0.0002$; *$p = 0.0184$) and EP300 (B, N.S. $p = 0.4808$; ***$p = 0.0006$) shows the specific downregulation of the respective mRNAs. (C) The effect of si-CREBBP and si-EP300 on the expression of CREB transcriptional coactivators in CASMCs. After the transfection of si-CREBBP, si-EP300, or si-NS, the expression of CREB transcriptional coactivators was analyzed by Western blotting. Gel images are representative results of three separate experiments. The transfection with si-CREBBP and si-EP300 under the experimental condition markedly attenuated the expression of the respective proteins.

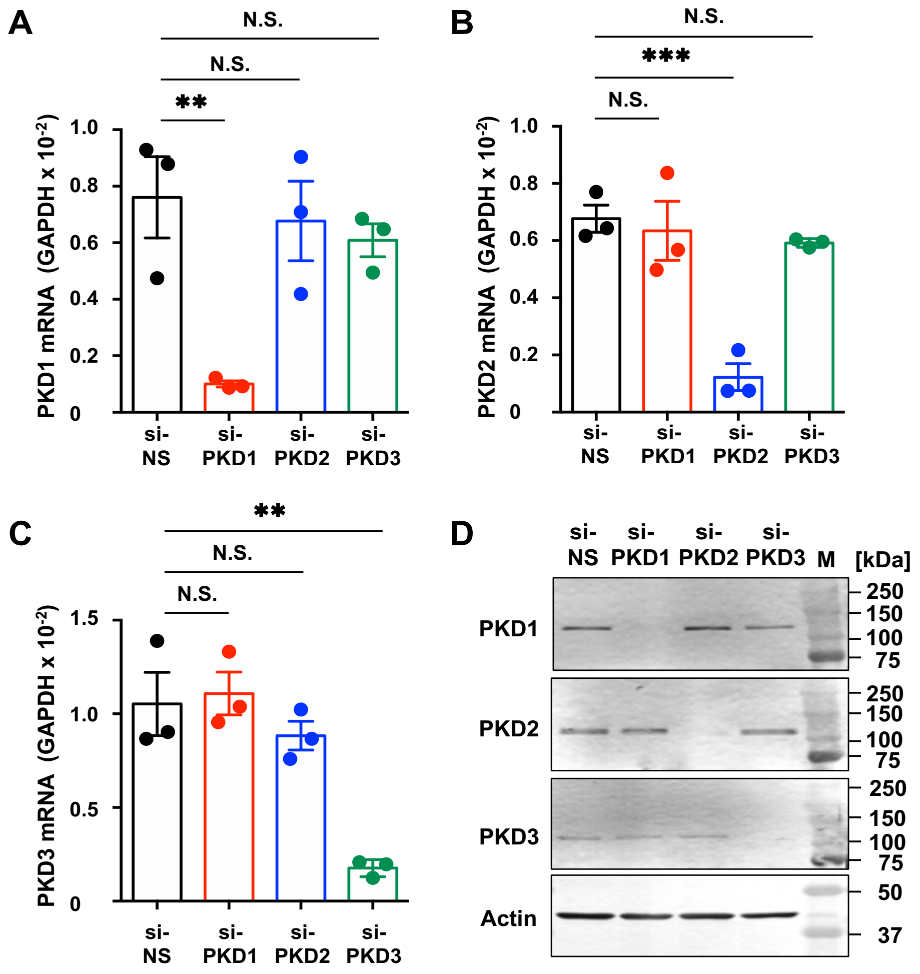

Figure S5. Expression of protein kinase D (PKD1-3) in CASMCs and their siRNA specificity.

(A–C) CASMCs were transfected with si-PKD1, si-PKD2, si-PKD3, or si-NS, and subsequent mRNA analysis was performed to assess the specificity of si-OGR1 for OGR1 expression. The expression of PKD1–3 mRNAs was measured by the RT-qPCR method. Results are expressed as relative ratios to GAPDH mRNA expression. Error bars represent the mean ± SEM (n = 3). Comparisons of si-PKD1, si-PKD2, or si-PKD3 vs. si-NS were assessed using a one-way ANOVA, followed by the Tukey test. The effect of si-PKD1–3 was significantly different than that of si-NS. The mRNA expression patterns of PKD1 (A, ***p* = 0.0055; N.S. *p* = 0.8958; N.S. *p* = 0.6302), PKD2 (B, N.S. *p* = 0.9272; ***p* = 0.0006; N.S. *p* = 0.6615), and PKD3 (C, N.S. *p* = 0.9684; N.S. *p* = 0.5843; ***p* = 0.0012) demonstrate specific downregulation of the respective mRNAs under the experimental condition. (D) The effect of siRNA for PKD1–3 on the expression of PKD1–3 proteins in CASMCs. After transfection of si-PKD1, si-PKD2, si-PKD3, or si-NS, the expression of PKD1-3 proteins was analyzed by Western blotting. Gel images are representative results of three separate experiments. Transfection with si-PKD1, si-PKD2 and si-PKD3 under the experimental condition markedly attenuated the expression of the respective proteins.

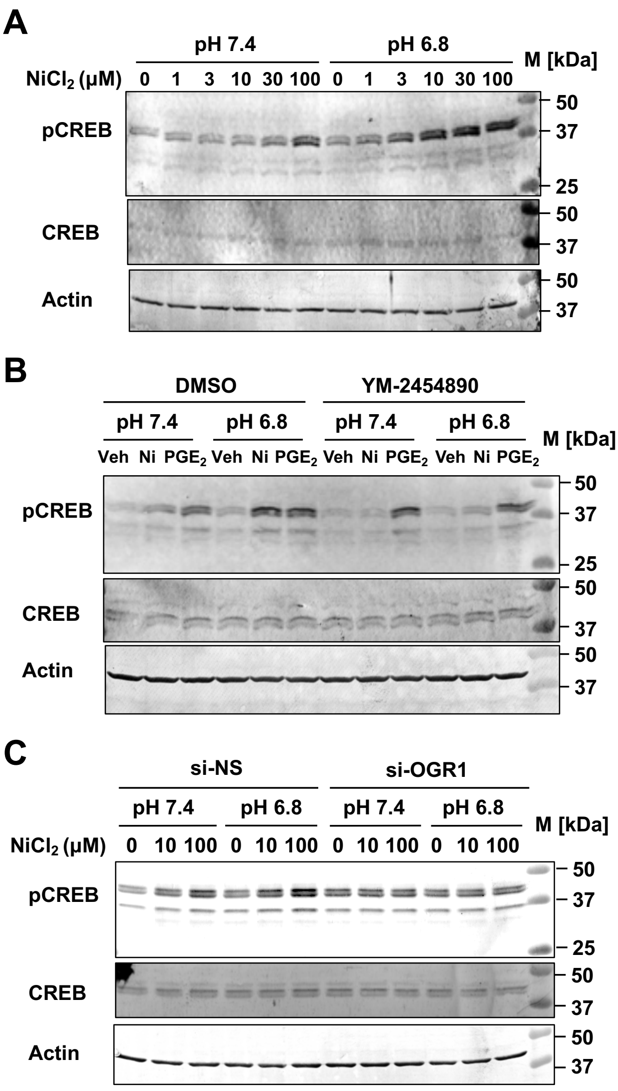

Figure S6. Subtle extracellular acidification increases for the phosphorylation of CREB in response to $NiCl_2$.

(A) After serum starvation for 8 hrs, CASMCs were stimulated at 37°C for 10 min by $NiCl_2$ (0–300 μM) in RPMI-1640-HEPES (pH 7.4–6.8)-0.1% BSA. The p-CREB and CREB together with the actin contents, were measured in cell lysate by Western blotting as described in the Experimental procedures section. Gel images are representative results of two separate experiments. (B) The effect of YM-254890 on CREB activation by induced $NiCl_2$. Serum-starved CASMCs were pretreated with YM-254890 (100 nM) or DMSO at 37°C for 30 min in RPMI-1640-0.1% BSA. The cells were stimulated for 10 min by $NiCl_2$ (Ni, 300 μM), PGE_2 (PE_2, 1 μM), or the vehicle (Veh) in RPMI-1640-HEPES (pH 7.4–6.8)-0.1% BSA containing YM-25489 or DMSO. Gel images are representative results of two separate experiments. (C) The effect of si-OGR1 on the CREB activation by induced $NiCl_2$. After serum starvation, siRNA-transfected CASMCs were incubated at 37°C for 10 min in RPMI-1640-HEPES (pH 7.4–6.8)-0.1% BSA with $NiCl_2$ (0–300 μM). Gel images are representative results of two separate experiments.
